# Gamma–distributed transmission memory in a reaction–diffusion epidemic model with nonlinear incidence

**DOI:** 10.1371/journal.pone.0360508

**Published:** 2026-10-14

**Authors:** Martin Anokye, Luca Guerrini, John Amoah Mensah, Michael Fosu Ofori, Stefania Ragni

**Affiliations:** 1 Department of Mathematics, University of Cape Coast, Cape Coast, Ghana; 2 Department of Management, Polytechnic University of Marche, Ancona, Italy; 3 Department of Computer Science, Sunyani Technical University, Sunyani, Ghana; 4 Department of Statistical Sciences, Kumasi Technical University, Kumasi, Ghana; 5 Department of Economics and Management, University of Ferrara, Ferrara, Italy; Fred Hutch Cancer Center: Fred Hutchinson Cancer Center, UNITED STATES OF AMERICA

## Abstract

We extend the delayed reaction–diffusion SI model with nonlinear incidence proposed by Song et al. by replacing the fixed transmission lag with weak and strong gamma-distributed memory kernels. Because the model has no exposed compartment, the memory term is interpreted phenomenologically as a distributed timing of transmission-related effects of past infectious states rather than as an explicit latent-stage mechanism. Using the same parameter set adopted by Song et al., we show that the distributed-memory models have a stability structure that is qualitatively different from that of the discrete-delay model. For the spatially homogeneous mode, both gamma kernels exhibit a small stabilising spectral Hopf crossing at a mean memory time near 0.08. The discrete-delay model has a comparable small stabilising crossing but, in addition, a much larger destabilising crossing near τ=1.335. Consequently, at the nominal values 1.2 and 1.6 used in the reference simulations, the gamma models converge to the selected endemic equilibrium, whereas the discrete-delay model crosses into an oscillatory regime. The first spatial mode is different: for the gamma formulations its modal constant term is negative for every positive memory rate, so this mode is unstable for every positive mean-memory value. We therefore interpret this behavior as a memory-independent spatial-mode instability, distinct from both a memory-induced Hopf crossing and a classical Turing instability.

## Introduction

The classical Kermack–McKendrick model [2] remains the basic compartmental framework for describing epidemic transmission. Since then, many extensions have been introduced in order to include features that are absent from the original formulation, such as nonlinear infection mechanisms, incubation delays and spatial movement. In particular, reaction–diffusion epidemic models combine disease dynamics with individual mobility, and therefore provide a natural setting for recurrent outbreaks, spatially heterogeneous equilibria and spatiotemporal patterns [3,4]. A first important extension concerns the incidence law. Liu, Levin and Iwasa [5] and Liu, Hethcote and Levin [6] showed that power-law incidence terms of the form $\beta S^{p}I^{q}$ can produce behaviour that is not present under bilinear incidence. Capasso and Serio [7] introduced a saturated incidence rate to describe inhibition effects, while Xiao and Ruan [8] proved that non-monotone incidence may generate limit cycles. A broad account of incidence functions and threshold dynamics is given by Hethcote [9], and the role of nonlinear incidence in global stability theory is discussed by Korobeinikov [10]. A second extension is the inclusion of a time delay. A fixed transmission lag leads to a discrete-delay model in which the infection term depends on the point value of the infected population at a past time. This changes the characteristic equations into quasipolynomials and may produce stability switches and Hopf bifurcations. Such mechanisms were analysed by Beretta and Takeuchi [11,12], Cooke and van den Driessche [13], and Ruan and Wei [14]. For epidemic models with nonlinear incidence, related stability results were obtained by McCluskey [15,16] and by Xu and Ma [17].

A fixed delay is mathematically convenient, but biologically restrictive: individuals do not all share the same transmission-related response time. Distributed-delay models replace the single value I(t−τ) by a weighted average of past infected densities [18,19]. When the weight is a gamma kernel, the linear chain trick converts the integro-differential equation into a finite-dimensional system, which makes the stability analysis more tractable [20]. Gamma and distributed-delay epidemic models have been studied, among others, by Forde and Nelson [21], Yuan and Bélair [22], Huang, Takeuchi, Ma and Wei [23], and Enatsu, Nakata and Muroya [24]. The relationship with the most closely related spatial and Erlang-delay literature is worth making explicit. Zhao, Wang and Zhang [25] studied a reaction–diffusion epidemic model with a general distributed delay, while Lamrani Alaoui et al. [26] analysed a diffusive SEIR model with distributed delay and nonlinear incidence. In a complementary nonspatial setting, Andò, Breda and Gava [27] gave a rigorous Erlang-delay/linear-chain analysis in epidemiology. These studies establish that distributed delays and Erlang chains are well motivated in epidemic dynamics, but they do not provide the mode-by-mode comparison developed here between the discrete-delay model of Song et al. [1] and weak/strong gamma transfer operators in the same nonlinear SI reaction–diffusion framework. The relation between discrete and distributed delays, especially in terms of stability switches, was already emphasised by Cooke and Grossman [28]. When diffusion is added, the temporal stability problem separates into spatial modes. This modal structure is essential because a homogeneous equilibrium can lose stability either through a homogeneous oscillation or through a spatially heterogeneous mode. Pattern formation and diffusion-driven instabilities are classical topics in spatial population dynamics [29], and the parabolic background is described in Pao [30]. In epidemic and related reaction–diffusion systems, stability and bifurcation analyses have been developed by Allen, Bolker, Lou and Nevai [31], Yi, Wei and Shi [32], Zuo and Wei [33], Sun [34], Du, Niu and Wei [35], and Cai and Wang [36]. Recent work has further emphasized that spatial epidemic and reaction–diffusion dynamics can be strongly altered by network topology, higher-order interactions and time-dependent spatial organization; representative examples include the West Nile virus model of Zhu and Zheng [37], the temporal-network Turing analysis of Shi and Zhu [38], and the network epidemic analysis of Dai et al. [39]. These studies do not use the same gamma-memory mechanism considered here, but they provide recent context for the broader question of how temporal and spatial structure modifies threshold and pattern-forming behavior. The specific contribution of the present paper is to compare, mode by mode, a fixed discrete transmission lag with two gamma-distributed memory operators while retaining the nonlinear SI reaction–diffusion structure of Song et al. [1].

Song, Ding, Feng and Shi [1] studied a reaction–diffusion SI model with nonlinear incidence $\beta SI^{2}$ and a discrete transmission delay τ. In their formulation the delay contributes the factor $e^{-\lambda \tau }$ to each modal characteristic equation, so spectral Hopf thresholds are obtained through trigonometric relations involving cos(ωτ) and sin(ωτ). Their numerical section uses specific values of τ to illustrate the transition from convergence to recurrent oscillations. The present paper keeps the same epidemiological structure and the same nonlinear incidence, but replaces the point delay with two gamma-distributed memories: the weak (exponential) kernel and the strong (second-order gamma) kernel. This produces a new model rather than a reparametrisation of the reference one. The transfer factor is no longer the pure phase shift $e^{-\lambda \tau }$, but the rational filter α/(λ+α) for the weak kernel and $\alpha^{2}/(\lambda +\alpha {)}^{2}$ for the strong kernel. As a consequence, the modal characteristic equations become algebraic polynomials, and their stability can be read through Routh–Hurwitz determinants. The main contribution is threefold. First, we derive the weak and strong gamma extensions and show that the endemic equilibrium is unchanged while the linearised dynamics are modified. Second, we obtain complete modal stability and Hopf conditions: a cubic criterion for the weak kernel and a quartic criterion for the strong kernel. Third, using the parameter set of Song et al. [1], we compare the new thresholds with the reference discrete-delay thresholds and interpret the simulations in detail. The comparison shows that distributed memory stabilises the homogeneous mode at a small mean memory time, whereas the reference discrete delay destabilises it at a much larger delay. It also shows that the first spatial mode is already unstable for positive gamma-memory rate, so its growth is a memory-independent spatial-mode instability rather than a new memory-induced spectral Hopf phenomenon.

The paper is organised as follows. Section 2 introduces the reference system and the gamma-distributed-delay extensions. Section 3 gives the endemic equilibrium and the linearisation. Section 4 derives the modal characteristic equations. Section 5 proves the Routh–Hurwitz stability and Hopf criteria. Section 6 computes the thresholds for the reference parameter set. Section 7 discusses the simulations through a continuous sequence of explanatory comparisons, and Sections 8–10 compare the models, discuss the implications and draw the conclusions.

## The reference model and the distributed-delay extension

We begin by recalling the benchmark discrete-delay system by Song et al. [1]. This formulation fixes the notation and provides the model against which the distributed-memory extensions are compared. The model is


$$\begin{array}{c} \left\{ \begin{array}{cc} \frac{\partial S}{\partial t} & =A-\beta S(x,t)I^{2}(x,t-\tau )-dS(x,t)+d_{1}\frac{\partial^{2}S}{\partial x^{2}},\\ \frac{\partial I}{\partial t} & =\beta S(x,t)I^{2}(x,t-\tau )-(\mu +d)I(x,t)+d_{2}\frac{\partial^{2}I}{\partial x^{2}}, \end{array}\right.\;x\in (0,\pi ),\;t>0, \end{array}$$

with homogeneous Neumann boundary conditions


$$\begin{array}{c} S_{x}(0,t)=S_{x}(\pi ,t)=I_{x}(0,t)=I_{x}(\pi ,t)=0. \end{array}$$ (1)

Here *S*(*x*,*t*) is the susceptible density and *I*(*x*,*t*) is the infected density. The parameters *A* (recruitment), *d* (natural death rate), μ (disease-induced removal rate), β (incidence coefficient) and $d_{1},d_{2}$ (diffusion coefficients) are all positive. The parameter τ>0 is a discrete transmission delay. We now replace the point delay by an averaged memory term. This step identifies the common distributed-delay structure before the two specific gamma kernels are introduced. Let K:[0,∞)→[0,∞) satisfy $\int_{0}^{\infty }K(s)\;ds=1$. We replace the point delay by


$$\begin{array}{c} Z(x,t)=\int_{0}^{\infty }K(s)I(x,t-s)\;ds, \end{array}$$

and set the incidence term to $\beta S(x,t)Z^{2}(x,t)$. The distributed-delay model is


$$\begin{array}{c} \left\{ \begin{array}{cc} S_{t} & =A-\beta SZ^{2}-dS+d_{1}S_{xx},\\ I_{t} & =\beta SZ^{2}-(\mu +d)I+d_{2}I_{xx}, \end{array}\right. \end{array}$$

with boundary conditions (1). We now choose two kernels that are both biologically interpretable and analytically tractable. They are written as local systems by means of the linear chain trick, so that the subsequent linearisation can be carried out using ordinary modal polynomials. The first admissible memory law is the weak gamma kernel. It converts the distributed history into one additional memory variable and therefore leads to the simplest local extension of the reference system. The weak kernel $K_{w}(s)=\alpha e^{-\alpha s}$ (α>0) has mean $T_{w}=1/\alpha$. Defining $V(x,t)=\int_{0}^{\infty }\alpha e^{-\alpha s}I(x,t-s)\;ds$ and differentiating gives $V_{t}=\alpha (I-V)$, so the weak gamma model is


$$\begin{array}{c} \left\{ \begin{array}{cc} S_{t} & =A-\beta SV^{2}-dS+d_{1}S_{xx},\\ I_{t} & =\beta SV^{2}-(\mu +d)I+d_{2}I_{xx},\\ V_{t} & =\alpha (I-V). \end{array}\right. \end{array}$$

The second memory law is the strong gamma kernel. It represents a two-stage transmission memory and introduces two auxiliary variables through the linear chain construction. The strong kernel $K_{s}(s)=\alpha^{2}se^{-\alpha s}$ (α>0) has mean $T_{s}=2/\alpha$. Introducing $V_{1}=\int_{0}^{\infty }\alpha e^{-\alpha s}I\;ds$ and $V_{2}=\int_{0}^{\infty }\alpha^{2}se^{-\alpha s}I\;ds$, the linear chain trick gives $\left(V_{1}{)}_{t}=\alpha (I-V_{1}\right)$ and $\left(V_{2}{)}_{t}=\alpha (V_{1}-V_{2}\right)$, so the strong gamma model is


$$\begin{array}{c} \left\{ \begin{array}{cc} S_{t} & =A-\beta SV_{2}^{2}-dS+d_{1}S_{xx},\\ I_{t} & =\beta SV_{2}^{2}-(\mu +d)I+d_{2}I_{xx},\\ (V_{1}{)}_{t} & =\alpha (I-V_{1}),\\ (V_{2}{)}_{t} & =\alpha (V_{1}-V_{2}). \end{array}\right. \end{array}$$

The strong kernel gives a more concentrated memory than the weak kernel for the same mean time. Thus the weak kernel represents a memory with maximal weight at the present and exponential decay into the past, whereas the strong kernel represents a two-stage memory whose weight is concentrated away from zero. Biologically, these kernels should be read as alternative assumptions on the timing and dispersion of the transmission contribution associated with past infectious states. The weak kernel allows the strongest weight immediately after the present and then decays monotonically, whereas the strong kernel suppresses zero-lag weight and concentrates influence around a positive time. Since the model is an SI system and contains no explicit exposed compartment, we use the expression transmission memory only as phenomenological shorthand; the variables *V*, *V*_1_ and *V*_2_ are memory filters, not latent or exposed compartments. Increasing the rate α shortens the mean memory time, while increasing $T_{w}=1/\alpha$ or $T_{s}=2/\alpha$ lengthens it. In what follows we therefore state explicitly whether a result concerns α or the mean memory time *T*.

## Equilibrium and linearisation

At a homogeneous equilibrium, every memory variable equals the constant infected density. Thus the equilibrium equations are identical to those of the reference model [1]:


$$\begin{array}{c} A-\beta S^{*}(I^{*}{)}^{2}-dS^{*}=0,\;\beta S^{*}(I^{*}{)}^{2}-(\mu +d)I^{*}=0. \end{array}$$

If $A^{2}\beta >4d(\mu +d{)}^{2}$, the equilibrium equations have two positive endemic branches. They may be written as


$$\begin{array}{c} I_{\pm }=\frac{A\beta \pm \sqrt{A^{2}\beta^{2}-4\beta d(\mu +d{)}^{2}}}{2\beta (\mu +d)},\;S_{\pm }=\frac{\mu +d}{\beta I_{\pm }}. \end{array}$$

To remain directly comparable with Song et al. [1], all subsequent calculations in this paper are carried out for the upper-infected/lower-susceptible branch $\left(S_{+},I_{+}\right)$. We denote this branch by $\left(S^{*},I^{*}\right)$ and equivalently write


$$\begin{array}{c} S^{*}=\frac{A\beta -\sqrt{A^{2}\beta^{2}-4\beta d(\mu +d{)}^{2}}}{2d\beta },\;I^{*}=\frac{2d(\mu +d)}{A\beta -\sqrt{A^{2}\beta^{2}-4\beta d(\mu +d{)}^{2}}}. \end{array}$$ (2)

For the reference parameter set used below, the second branch is $\left(S_{-},I_{-})=(0.5707106781,0.1533176150\right)$ and is already a saddle in the homogeneous zero-memory linearization (eigenvalues approximately −0.4601 and 1.5079). Hence the stability and crossing results reported below refer specifically to $\left(S^{*},I^{*}\right)$, not to both endemic branches. Setting $a_{11}=-\beta (I^{*}{)}^{2}-d$, $a_{13}=-2\beta S^{*}I^{*}$, $a_{21}=\beta (I^{*}{)}^{2}$, $a_{22}=-(\mu +d)$, $a_{23}=2\beta S^{*}I^{*}$, the linearised weak and strong systems around the endemic equilibrium $\left(S^{*},I^{*},I^{*},...\right)$ are respectively


$$\begin{array}{c} \left\{ \begin{array}{cc} u_{t} & =a_{11}u+a_{13}w+d_{1}u_{xx},\\ v_{t} & =a_{21}u+a_{22}v+a_{23}w+d_{2}v_{xx},\\ w_{t} & =\alpha (v-w), \end{array}\right. \end{array}$$ (3)

and


$$\begin{array}{c} \left\{ \begin{array}{cc} u_{t} & =a_{11}u+a_{13}w_{2}+d_{1}u_{xx},\\ v_{t} & =a_{21}u+a_{22}v+a_{23}w_{2}+d_{2}v_{xx},\\ (w_{1}{)}_{t} & =\alpha (v-w_{1}),\\ (w_{2}{)}_{t} & =\alpha (w_{1}-w_{2}), \end{array}\right. \end{array}$$ (4)

where (*u*,*v*,*w*) and $\left(u,v,w_{1},w_{2}\right)$ are perturbations from equilibrium. The equilibrium calculation shows that the distributed memory does not change the endemic state. The important changes appear only after linearisation, because perturbations are filtered differently by the weak and strong kernels. We therefore pass from the linearised systems to the modal characteristic equations.

## Modal characteristic equations

We insert modal perturbations $e^{\lambda t}\cos(kx)$, k=0,1,2,..., and define


$$\begin{array}{c} p_{k}=d_{1}k^{2}-a_{11},\;q_{k}=d_{2}k^{2}-a_{22}. \end{array}$$

We first record the modal equation of the reference discrete-delay model [1]. This equation is not reanalysed in detail here, but it provides the benchmark for the comparison with the gamma models.


$$\begin{array}{c} (\lambda +p_{k})(\lambda +q_{k})-[a_{23}(\lambda +p_{k})+a_{13}a_{21}]e^{-\lambda \tau }=0. \end{array}$$ (5)

This transcendental equation is a quasipolynomial and requires trigonometric Hopf analysis. It is recalled only as the reference against which the gamma models are compared. We next derive the modal equation for the weak gamma model. The memory equation turns the distributed feedback into a rational transfer factor and gives a cubic characteristic polynomial. From the memory equation of (3), w=[α/(λ+α)]v. Substitution gives


$$\begin{array}{c} (\lambda +\alpha )(\lambda +p_{k})(\lambda +q_{k})-\alpha [a_{23}(\lambda +p_{k})+a_{13}a_{21}]=0, \end{array}$$ (6)

which expands to the cubic


$$\begin{array}{c} \lambda^{3}+A_{1,k}^{\left(w\right)}\lambda^{2}+A_{2,k}^{\left(w\right)}\lambda +A_{3,k}^{\left(w\right)}=0, \end{array}$$ (7)

with


$$\begin{array}{cc} A_{1,k}^{\left(w\right)} & =\alpha +p_{k}+q_{k},\\ A_{2,k}^{\left(w\right)} & =p_{k}q_{k}+\alpha (p_{k}+q_{k})-\alpha a_{23},\\ A_{3,k}^{\left(w\right)} & =\alpha (p_{k}q_{k}-a_{23}p_{k}-a_{13}a_{21}). \end{array}$$ (8)

We then repeat the same calculation for the strong gamma model. The two-stage chain produces a squared rational transfer factor and therefore a quartic characteristic polynomial. From (4), $w_{2}=[\alpha /(\lambda +\alpha ){]}^{2}v$. Substitution gives


$$\begin{array}{c} (\lambda +\alpha {)}^{2}(\lambda +p_{k})(\lambda +q_{k})-\alpha^{2}[a_{23}(\lambda +p_{k})+a_{13}a_{21}]=0, \end{array}$$ (9)

which expands to the quartic


$$\begin{array}{c} \lambda^{4}+B_{1,k}^{\left(s\right)}\lambda^{3}+B_{2,k}^{\left(s\right)}\lambda^{2}+B_{3,k}^{\left(s\right)}\lambda +B_{4,k}^{\left(s\right)}=0, \end{array}$$ (10)

with


$$\begin{array}{cc} B_{1,k}^{\left(s\right)} & =p_{k}+q_{k}+2\alpha ,\\ B_{2,k}^{\left(s\right)} & =p_{k}q_{k}+2\alpha (p_{k}+q_{k})+\alpha^{2},\\ B_{3,k}^{\left(s\right)} & =2\alpha p_{k}q_{k}+\alpha^{2}(p_{k}+q_{k})-\alpha^{2}a_{23},\\ B_{4,k}^{\left(s\right)} & =\alpha^{2}(p_{k}q_{k}-a_{23}p_{k}-a_{13}a_{21}). \end{array}$$

**Remark 1**. Equations (5), (6) and (9) make the comparison explicit: the transfer factor changes from $e^{-\lambda \tau }$ (discrete) to α/(λ+α) (weak) to $\alpha^{2}/(\lambda +\alpha {)}^{2}$ (strong). These are fundamentally different operators; in particular, the Laplace-domain transfer function of the gamma memory is a low-pass rational filter, whereas the discrete delay introduces a pure phase shift.

## Stability and spectral Hopf-crossing analysis

The modal equations derived above reduce the local problem to finite-dimensional characteristic polynomials. We now apply the standard Routh–Hurwitz and spectral crossing criteria used in local dynamical-systems analysis [40,41]. The purpose of this section is not only to state stability conditions, but also to identify which determinant is responsible for a possible crossing of the imaginary axis. We start with the weak kernel, because its cubic equation gives the most transparent stability test. The Routh–Hurwitz determinant identifies the possible Hopf crossing in each spatial mode. For the cubic $\lambda^{3}+A_{1}\lambda^{2}+A_{2}\lambda +A_{3}=0$, the Routh–Hurwitz criterion requires *A*_1_ > 0, *A*_2_ > 0, *A*_3_ > 0 and $A_{1}A_{2}>A_{3}$.

**Theorem 1** (Weak gamma stability and spectral Hopf condition)**.** *Fix a Neumann mode* $k\in {\mathbb{N}}_{0}$*. The weak gamma model is locally asymptotically stable in the k-th mode if and only if*


$$\begin{array}{c} A_{1,k}^{\left(w\right)}>0,\;A_{2,k}^{\left(w\right)}>0,\;A_{3,k}^{\left(w\right)}>0,\;\Delta_{k}^{\left(w\right)}(\alpha )>0, \end{array}$$ (11)

*where* $\Delta_{k}^{\left(w\right)}(\alpha )=A_{1,k}^{\left(w\right)}A_{2,k}^{\left(w\right)}-A_{3,k}^{\left(w\right)}$*. A simple spectral Hopf crossing occurs when the first three conditions hold,* $\Delta_{k}^{\left(w\right)}=0$*, and* $d\Delta_{k}^{\left(w\right)}/d\alpha \neq 0$*. At the crossing, the Hopf frequency is* $\omega_{k,w}=\sqrt{A_{2,k}^{\left(w\right)}}$.

*Proof.* Routh–Hurwitz applied to (7) gives (11). When $\Delta_{k}^{\left(w\right)}=0$ with $A_{j,k}^{\left(w\right)}>0$ (*j* = 1,2,3), the cubic factors as $(\lambda +A_{1,k}^{\left(w\right)})(\lambda^{2}+A_{2,k}^{\left(w\right)})=0$, giving roots $-A_{1,k}^{\left(w\right)}$ and $\pm i\sqrt{A_{2,k}^{\left(w\right)}}$. The nonzero derivative ensures transversality. □

Substituting (8) into $\Delta_{k}^{\left(w\right)}=0$ gives the quadratic


$$\begin{array}{c} \alpha^{2}(p_{k}+q_{k}-a_{23})+\alpha \{(p_{k}+q_{k})(p_{k}+q_{k}-a_{23})+a_{23}p_{k}+a_{13}a_{21}\}+(p_{k}+q_{k})p_{k}q_{k}=0. \end{array}$$ (12)

Positive roots of (12) are candidate spectral crossing values because $\Delta_{k}^{\left(w\right)}=0$ is already imposed by that equation. A candidate is accepted as a simple spectral Hopf crossing only if $A_{1,k}^{\left(w\right)}$, $A_{2,k}^{\left(w\right)}$ and $A_{3,k}^{\left(w\right)}$ are positive and $d\Delta_{k}^{\left(w\right)}/d\alpha \neq 0$. Thus the strict inequality $\Delta_{k}^{\left(w\right)}>0$ in the stability condition (11) is not imposed at the crossing itself. This explicit separation resolves the apparent conflict between (11) and (12). We then pass to the strong kernel. The characteristic equation is now quartic, so the relevant Routh–Hurwitz determinant is different, but the logic remains the same. For the quartic $\lambda^{4}+B_{1}\lambda^{3}+B_{2}\lambda^{2}+B_{3}\lambda +B_{4}=0$, Routh–Hurwitz requires $B_{j}>0$ (j=1,...,4) and $B_{1}B_{2}B_{3}>B_{3}^{2}+B_{1}^{2}B_{4}$.

**Theorem 2** (Strong gamma stability and spectral Hopf condition)**.** *Fix a Neumann mode* $k\in {\mathbb{N}}_{0}$*. The strong gamma model is locally asymptotically stable in the k-th mode if and only if*


$$\begin{array}{c} B_{j,k}^{\left(s\right)}>0\;\;(j=1,2,3,4),\;\Delta_{k}^{\left(s\right)}(\alpha )>0, \end{array}$$ (13)

*where*


$$\begin{array}{c} \Delta_{k}^{\left(s\right)}(\alpha )=B_{1,k}^{\left(s\right)}B_{2,k}^{\left(s\right)}B_{3,k}^{\left(s\right)}-(B_{3,k}^{\left(s\right)}{)}^{2}-(B_{1,k}^{\left(s\right)}{)}^{2}B_{4,k}^{\left(s\right)}. \end{array}$$

*A simple spectral Hopf crossing occurs when* $B_{j,k}^{\left(s\right)}>0$ *(j = 1,2,3,4),* $\Delta_{k}^{\left(s\right)}=0$*, and* $d\Delta_{k}^{\left(s\right)}/d\alpha \neq 0$*. At such a crossing, the Hopf frequency is*


$$\begin{array}{c} \omega_{k,s}=\sqrt{\frac{B_{3,k}^{\left(s\right)}}{B_{1,k}^{\left(s\right)}}}. \end{array}$$ (14)

*Proof.* The Routh–Hurwitz criterion applied to (10) gives (13). At a Hopf crossing, let λ=iω with ω>0. Separating real and imaginary parts of (10):


$$\begin{array}{c} \omega^{4}-B_{2,k}^{\left(s\right)}\omega^{2}+B_{4,k}^{\left(s\right)}=0,\;-B_{1,k}^{\left(s\right)}\omega^{3}+B_{3,k}^{\left(s\right)}\omega =0. \end{array}$$

Since ω>0, the imaginary equation yields $\omega^{2}=B_{3,k}^{\left(s\right)}/B_{1,k}^{\left(s\right)}$, which gives (14). Substituting into the real equation:


$$\begin{array}{c} (\frac{B_{3,k}^{\left(s\right)}}{B_{1,k}^{\left(s\right)}}{)}^{2}-B_{2,k}^{\left(s\right)}\frac{B_{3,k}^{\left(s\right)}}{B_{1,k}^{\left(s\right)}}+B_{4,k}^{\left(s\right)}=0, \end{array}$$

which is equivalent to $\Delta_{k}^{\left(s\right)}=0$. Transversality follows from the nonzero derivative. □

**Remark 2**. The Hopf frequency for the strong kernel is $\omega_{k,s}=\sqrt{B_{3,k}^{\left(s\right)}/B_{1,k}^{\left(s\right)}}$, derived from the imaginary part of the characteristic equation. This should not be confused with the expression $\sqrt{B_{4,k}^{\left(s\right)}/B_{2,k}^{\left(s\right)}}$; the two quantities are not equal in general, and only the formula in (14) is correct.

The weak and strong criteria identify spectral Hopf crossings of the linearised characteristic roots. We do not infer a full nonlinear Hopf bifurcation solely from these conditions; such a claim would additionally require a nonlinear nondegeneracy calculation (for example, a first Lyapunov coefficient on the relevant center manifold). Accordingly, the numerical section uses the terms “spectral crossing” or “Hopf-type spectral crossing” unless referring to a nonlinear Hopf result already established for the reference model. The weak and strong criteria describe spectral crossings caused by the memory parameter. Before applying them to the reference parameter set, however, it is necessary to separate these memory-induced crossings from instabilities that are already present when the memory time is zero. The following diagnostic performs this separation mode by mode and is essential for interpreting the spatial modes correctly. The expression


$$\begin{array}{c} {𝒞}_{k}=p_{k}q_{k}-a_{23}p_{k}-a_{13}a_{21} \end{array}$$

appears as the core constant factor in both gamma models: $A_{3,k}^{\left(w\right)}=\alpha {𝒞}_{k}$ and $B_{4,k}^{\left(s\right)}=\alpha^{2}{𝒞}_{k}$.

**Proposition 1** (Memory-independent modal instability). *If* ${𝒞}_{k}<0$ *for some mode k, then the selected endemic equilibrium is unstable in that mode for every* α>0 *in both the weak and strong gamma models. At zero delay, the corresponding discrete-delay modal equation is also unstable. Therefore the gamma instability is not created by varying a positive memory parameter and cannot be classified as a memory-induced spectral Hopf crossing.*

*Proof.* For the weak gamma model, $A_{3,k}^{\left(w\right)}=\alpha {𝒞}_{k}<0$ for every α>0. Hence the cubic characteristic polynomial is negative at λ=0 and tends to +∞ as λ→+∞, so it has a positive real root. The same argument applies to the strong gamma quartic because $B_{4,k}^{\left(s\right)}=\alpha^{2}{𝒞}_{k}<0$ for every α>0. For the discrete-delay model at τ=0, evaluating the characteristic polynomial at λ=0 gives the same quantity ${𝒞}_{k}<0$, again implying a positive real root. □

**Remark 3**. The term “memory-independent spatial-mode instability” is sometimes reserved for the classical Turing mechanism, in which diffusion destabilises a homogeneous equilibrium that is stable without diffusion. We do not use that terminology here for *k = 1,* because for the reference parameter set the homogeneous mode is itself unstable in the zero-memory limit. We instead call the *k = 1* phenomenon a memory-independent spatial-mode instability: it is spatially heterogeneous, but it is not by itself evidence of a classical Turing instability.

## Numerical calculations with the reference parameter set

The parameter set of the reference paper [1] is


$$\begin{array}{c} d_{1}=6,\;d_{2}=1,\;A=1,\;\beta =32,\;\mu =1.8,\;d=1. \end{array}$$

The endemic equilibrium from (2) is


$$\begin{array}{c} S^{*}=0.4292893219,\;I^{*}=0.2038252422. \end{array}$$

The linearisation coefficients are


$$\begin{array}{cc} a_{11} & =-2.3294313393,\;a_{13}=-5.6000000000,\;a_{21}=1.3294313393,\\ a_{22} & =-2.8000000000,\;a_{23}=5.6000000000. \end{array}$$

We first evaluate the homogeneous mode *k* = 0. This mode describes spatially uniform perturbations and gives the threshold that is directly comparable with the homogeneous spectral Hopf threshold of the reference model. For *k* = 0,


$$\begin{array}{c} p_{0}=2.3294313393,\;q_{0}=2.8000000000,\;{𝒞}_{0}=0.9224077499>0. \end{array}$$

The coefficient $p_{0}+q_{0}-a_{23}=-0.4705686607<0$, so the zero-memory homogeneous mode is unstable. Increasing the mean memory time *T* (equivalently, decreasing the rate α) stabilises it. For the weak kernel, equation (12) has the positive root


$$\begin{array}{c} \alpha_{0,w}=12.4717466127,\;T_{0,w}=1/\alpha_{0,w}=0.0801812361,\;\omega_{0,w}=0.8084492080. \end{array}$$

For the strong kernel, $\Delta_{0}^{\left(s\right)}=0$ has the positive root


$$\begin{array}{c} \alpha_{0,s}=24.6848909027,\;T_{0,s}=2/\alpha_{0,s}=0.0810212447,\;\omega_{0,s}=0.8044866545. \end{array}$$

The numerical value of $\omega_{0,s}$ equals $\sqrt{B_{3,0}^{\left(s\right)}/B_{1,0}^{\left(s\right)}}$ evaluated at $\alpha_{0,s}$, confirming formula (14). The transversality conditions are also numerically nondegenerate. For the weak kernel,


$$\begin{array}{c} {d\Delta_{0}^{\left(w\right)}/d\alpha |}_{\alpha_{0,w}}=-8.55138, \end{array}$$

and for the strong kernel,


$$\begin{array}{c} {d\Delta_{0}^{\left(s\right)}/d\alpha |}_{\alpha_{0,s}}=-5.63651\times 10^{5}. \end{array}$$

Equivalently, the derivative of the spectral abscissa with respect to mean memory time is approximately −2.14221 (weak) and −2.08672 (strong) at the respective crossings. Thus each pair of imaginary roots crosses the imaginary axis transversally. Both gamma crossings are stabilising with respect to the mean memory time: for *T* > *T*_0_ the homogeneous mode is stable, whereas for *T* < *T*_0_ it is unstable. This behavior is analogous to the small stabilising crossing of the discrete-delay model, but differs from its additional large destabilising crossing near τ=1.335. For the discrete-delay model, the characteristic equation for *k* = 0 has imaginary roots at two values of τ. A small crossing near τ=0.08194 is stabilising (analogous to the gamma thresholds), and a large destabilising crossing occurs at


$$\begin{array}{c} \tau_{0,d}=1.3348431043,\;\omega_{0,d}=4.1776873277. \end{array}$$

The reference simulations use τ=1.2 (stable) and τ=1.6 (oscillatory) to straddle this second crossing. Table 1 summarizes the homogeneous-mode stability thresholds and the corresponding critical frequencies for the three delay formulations.

**Table 1:** Homogeneous-mode thresholds for the reference parameter set. For gamma kernels the threshold is expressed as mean memory time; for the discrete delay it is τ.

Model	Characteristic equation	Critical value	Frequency
Discrete delay	quasipolynomial	τ≃0.08194 (stab.)	0.80042
Discrete delay	quasipolynomial	τ≃1.33484 (destab.)	4.17769
Weak gamma	cubic	$T_{w}≃0.08018$ (stab.)	0.80845
Strong gamma	quartic	$T_{s}≃0.08102$ (stab.)	0.80449

Fig 1 gives the same comparison in graphical form and highlights the separation between the small stabilising thresholds and the large destabilising discrete-delay threshold.

**Fig 1:**
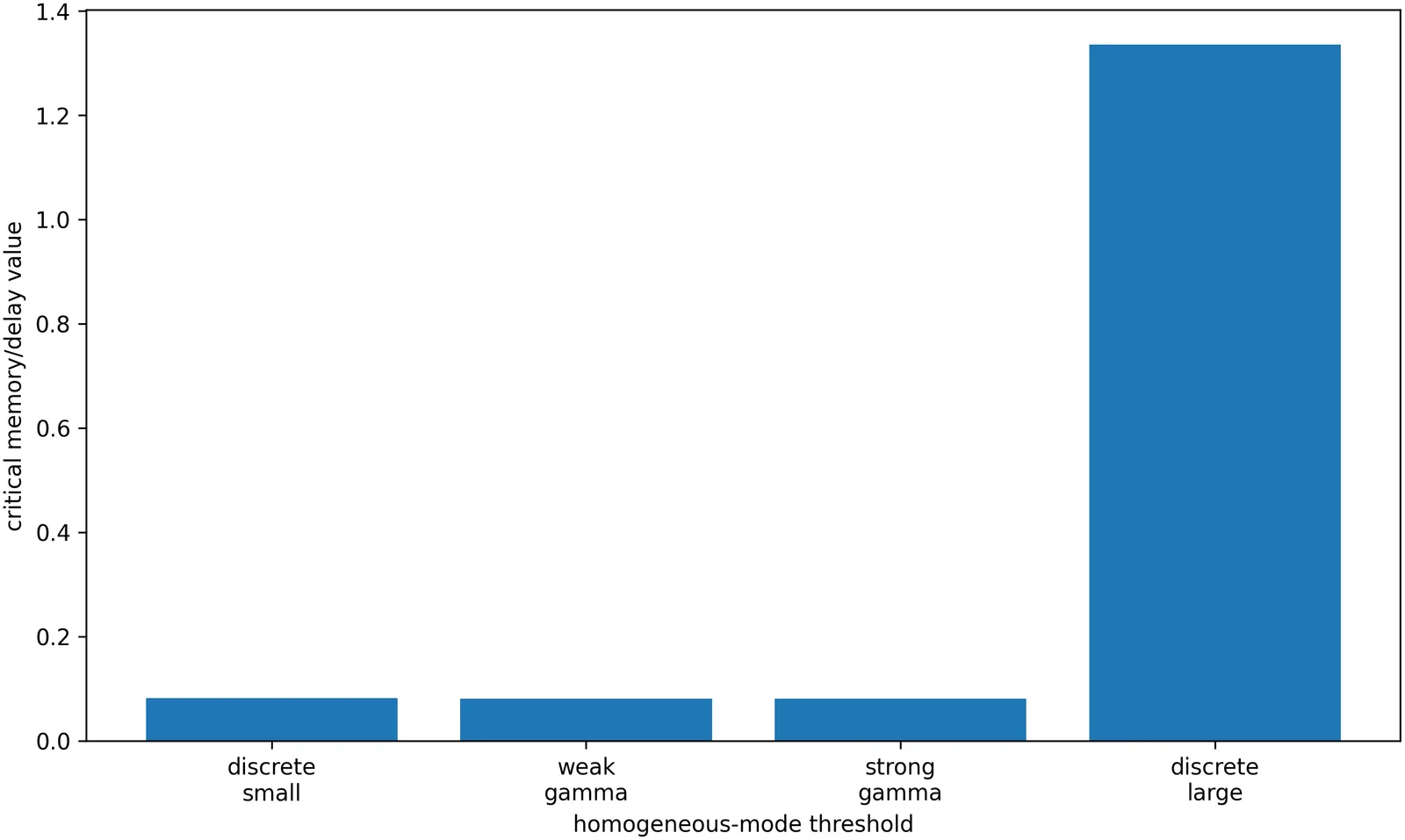
Graphical comparison of homogeneous-mode thresholds. The weak and strong gamma thresholds are close to the small stabilising discrete-delay threshold, whereas the large discrete-delay threshold is much farther away and has the opposite stability effect.

The key observation is that the gamma thresholds (T≈0.08) and the small discrete-delay threshold (τ≈0.082) are comparable and stabilising, while the large discrete-delay threshold (τ≈1.335) is destabilising and has no gamma analogue. This explains why at nominal values *T* = 1.2 and *T* = 1.6 the gamma models behave differently from the discrete-delay model. We next examine the first spatial mode *k* = 1. This mode is important because it reveals whether the observed spatial growth is caused by memory or by the reaction–diffusion balance itself.


$$\begin{array}{c} p_{1}=8.3294313393,\;q_{1}=3.8000000000,\;{𝒞}_{1}=-7.5481609108<0. \end{array}$$

By Proposition 1, the first spatial mode is already unstable at zero memory. The zero-delay positive eigenvalue is approximately $\lambda_{1,+}(0)=1.0022$. This is a memory-independent spatial-mode instability, not a memory-induced spectral Hopf phenomenon and not, by itself, a classical Turing instability. We finally establish analytically the stability of all higher spatial modes k≥2. The value *k* = 2 is reported first only for illustration. For *k* = 2, *p*_2_ = 26.3294313393, *q*_2_ = 6.8000000000, ${𝒞}_{2}=39.0401331070>0$. To prove the result for every higher mode, put $x=k^{2}\geq 4$. For the reference parameter set,


$$\begin{array}{c} p_{k}=6x+2.3294313393,\;q_{k}=x+2.8,\;{𝒞}_{k}=6x^{2}-14.4705686607x+0.9224077500. \end{array}$$

Since ${𝒞}_{k}^{\prime }(x)=12x-14.4705686607>0$ for x≥4,


$$\begin{array}{c} {𝒞}_{k}\geq {𝒞}_{k}{|}_{x=4}=39.0401331072>0. \end{array}$$

Moreover, $p_{k}+q_{k}-a_{23}=7x-0.4705686607>0$ for x≥4, so all coefficients of the weak cubic are positive. Furthermore,


$$\begin{array}{c} \begin{array}{cc} \Delta_{k}^{\left(w\right)}=\; & (7x-0.4705686607)\alpha^{2}+(49x^{2}+66.2120387502x+3.1862503645)\alpha \\ & +(42x^{3}+164.6826074109x^{2}+143.7799588651x+33.4562427207). \end{array} \end{array}$$

Every coefficient in this polynomial is strictly positive for x≥4, hence $\Delta_{k}^{\left(w\right)}>0$ for every α>0. For the strong kernel, the quartic coefficients $B_{j,k}^{\left(s\right)}$ are positive by the same inequalities, and the Hurwitz determinant can be written as


$$\begin{array}{c} \Delta_{k}^{\left(s\right)}=\sum_{m=1}^{5}c_{m}(x)\alpha^{m}, \end{array}$$

where


$$\begin{array}{cc} c_{5}(x)=\; & 14x-0.9411373214,\\ c_{4}(x)=\; & 196x^{2}+304.0481550008x+10.1098169581,\\ c_{3}(x)=\; & 854x^{3}+2625.9832433978x^{2}+1936.3349708x+297.0128736328,\\ c_{2}(x)=\; & 1176x^{4}+6884.0574725076x^{3}+9169.8132586922x^{2}+4367.8439644511x+646.4330092337,\\ c_{1}(x)=\; & 504x^{5}+3583.063521432x^{4}+8573.8110198717x^{3}+8050.5868445499x^{2}\\ & +3155.580831999x+436.4305136181. \end{array}$$

For x≥4, $c_{5}(x)\geq 55.0588626786>0$, while $c_{1}(x),...,c_{4}(x)$ are polynomials in *x* with strictly positive coefficients. Therefore $c_{m}(x)>0$ for every m=1,...,5 and every x≥4, so $\Delta_{k}^{\left(s\right)}>0$ for every α>0. Consequently, both gamma models satisfy the complete Routh–Hurwitz conditions for every k≥2 and every α>0. Thus no spectral Hopf crossing occurs in these higher modes.

## Numerical simulations

The numerical simulations are organised to answer a specific modelling question: does a gamma-distributed transmission memory reproduce the same instability mechanism found in the discrete-delay reference model, or does it lead to a different qualitative behaviour? In all cases the selected endemic equilibrium and the parameter values are those reported above; only the memory law is changed.

For reproducibility, Figs 2–6 are simulations of the spatially homogeneous reduced systems (no diffusion terms are active because *S* and *I* are independent of *x*). We used a classical fourth-order Runge–Kutta method with fixed step Δt=0.005 on 0≤t≤80. The discrete-delay equation was integrated by the method of steps, with linear interpolation of the stored delayed state. The common initialization was $S(0)=S^{*}$ and $I(0)=0.98I^{*}=0.1997487373$. For the discrete-delay model the history was constant, $I(t)=0.98I^{*}$ for t≤0. To impose the equivalent initial memory state in the gamma models, we set *V*(0)=*I*(0) for the weak kernel and $V_{1}(0)=V_{2}(0)=I(0)$ for the strong kernel. Thus differences in the transients are not caused by inconsistent initialization. Figs 7–8 are spectral calculations from the characteristic polynomials, not time integrations. Fig 9 is a linear modal reconstruction, $I(x,t)=I^{*}+\varepsilon e^{\lambda_{1,+}t}\cos x$, with ε=0.002 and $\lambda_{1,+}=1.0022$; it is not presented as a nonlinear full-PDE simulation.

**Fig 2:**
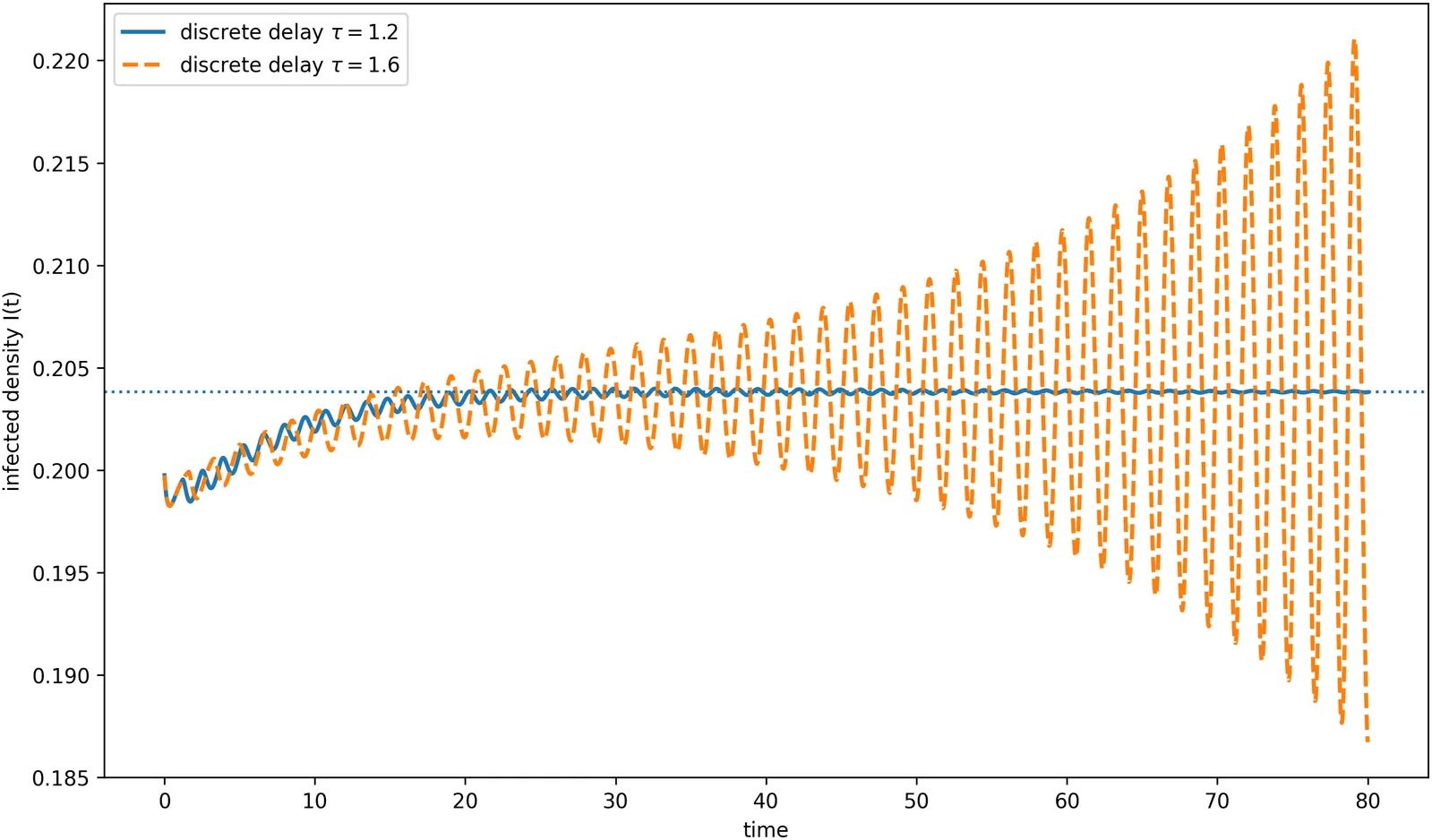
Homogeneous discrete-delay simulations. Infected density *I*(*t*) for the Song et al. parameter set *d*_1_ = 6, *d*_2_ = 1, *A* = 1, β=32, μ=1.8, *d* = 1, with $S(0)=S^{*}$ and constant history $I(t)=0.98I^{*}$ for t≤0. Solid: τ=1.2, inside the stable window. Dashed: τ=1.6, beyond the large destabilising spectral crossing $\tau_{0,d}≃1.335$. The dotted horizontal line is $I^{*}=0.2038252422$.

**Fig 3:**
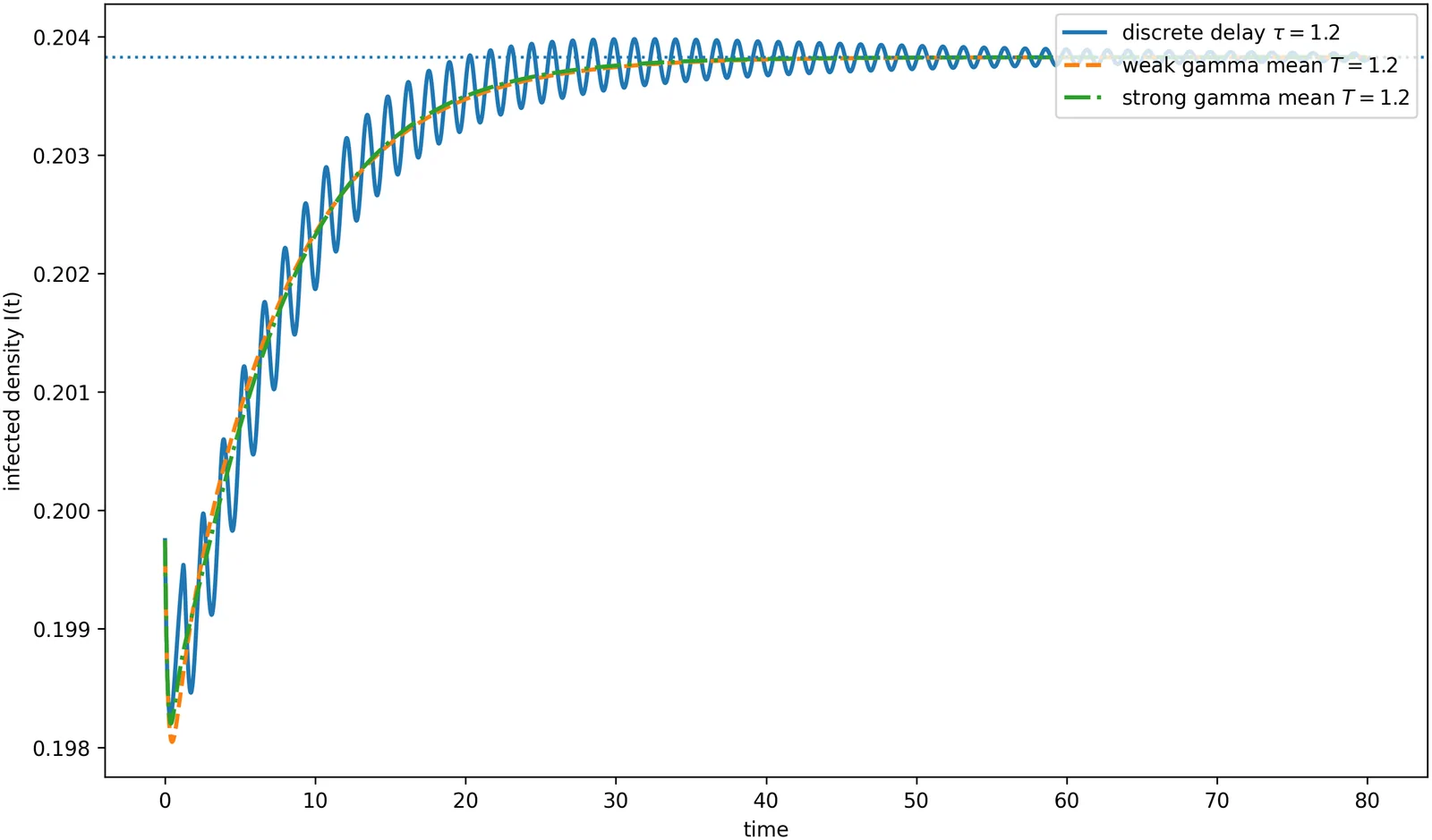
Homogeneous comparison at nominal value 1.2. Infected density *I*(*t*) for *d*_1_ = 6, *d*_2_ = 1, *A* = 1, β=32, μ=1.8, *d* = 1 and equivalent initial memory states. Solid: discrete delay τ=1.2. Dashed: weak gamma with mean $T_{w}=1.2$ ($\alpha =1/T_{w}$). Dash-dotted: strong gamma with mean $T_{s}=1.2$ ($\alpha =2/T_{s}$). All converge to $I^{*}$ (dotted horizontal line), although the transient filters differ.

**Fig 4:**
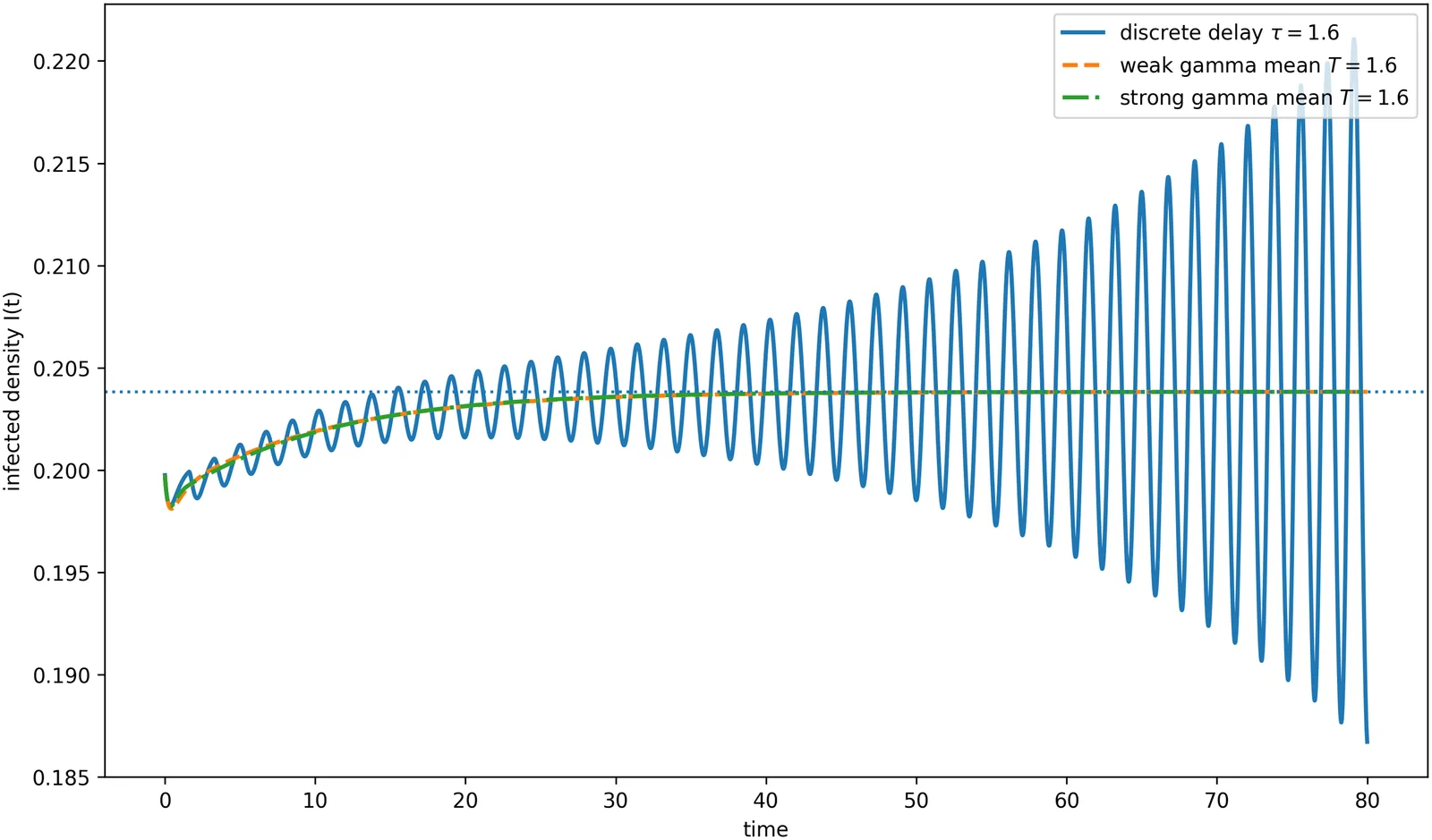
Homogeneous comparison at nominal value 1.6. Infected density *I*(*t*) for *d*_1_ = 6, *d*_2_ = 1, *A* = 1, β=32, μ=1.8, *d* = 1 and equivalent initial memory states. Solid: discrete delay τ=1.6, beyond the large destabilising spectral crossing. Dashed: weak gamma with $T_{w}=1.6$. Dash-dotted: strong gamma with $T_{s}=1.6$. The gamma trajectories converge to $I^{*}$ (dotted horizontal line), whereas the discrete-delay oscillations grow.

**Fig 5:**
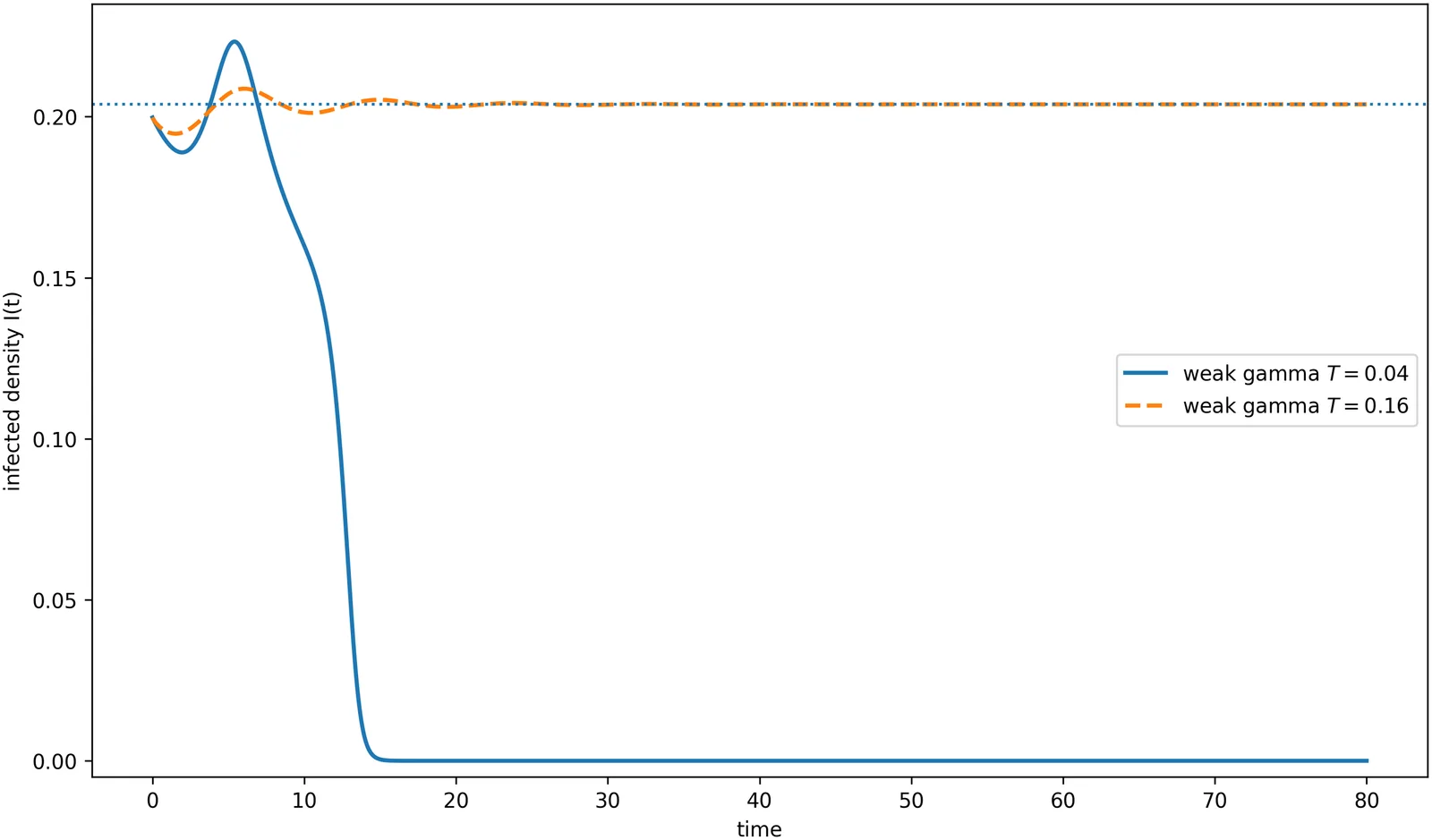
Homogeneous weak-gamma simulations. Infected density *I*(*t*) for *d*_1_ = 6, *d*_2_ = 1, *A* = 1, β=32, μ=1.8, *d* = 1, with $S(0)=S^{*}$, $I(0)=V(0)=0.98I^{*}$. Solid: $T_{w}=0.04<T_{0,w}$, for which the selected endemic equilibrium is unstable. Dashed: $T_{w}=0.16>T_{0,w}$, for which the trajectory converges to $I^{*}$ (dotted horizontal line).

**Fig 6:**
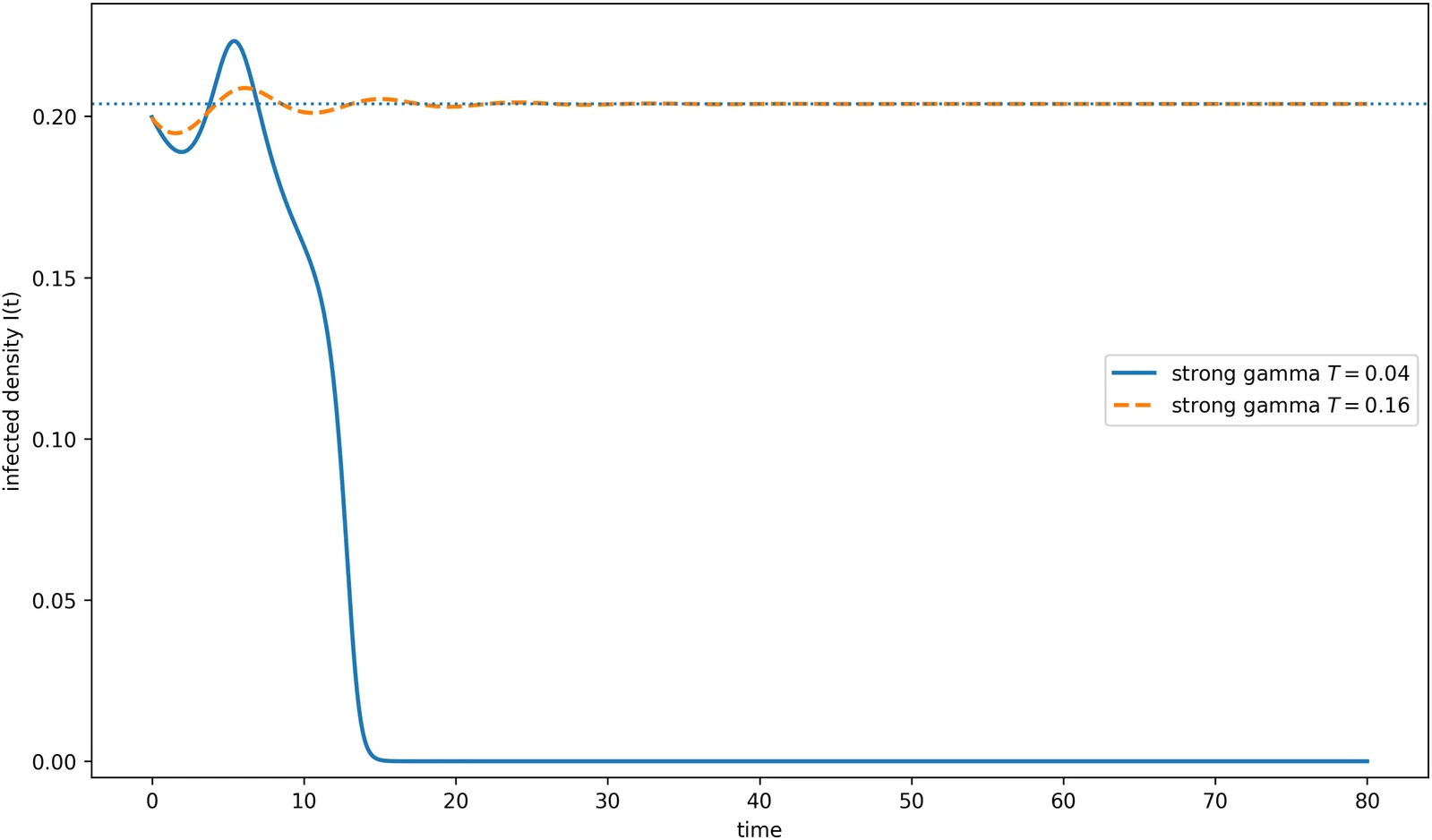
Homogeneous strong-gamma simulations. Infected density *I*(*t*) for *d*_1_ = 6, *d*_2_ = 1, *A* = 1, β=32, μ=1.8, *d* = 1, with $S(0)=S^{*}$ and $I(0)=V_{1}(0)=V_{2}(0)=0.98I^{*}$. Solid: $T_{s}=0.04<T_{0,s}$. Dashed: $T_{s}=0.16>T_{0,s}$ and converges to $I^{*}$ (dotted horizontal line).

**Fig 7:**
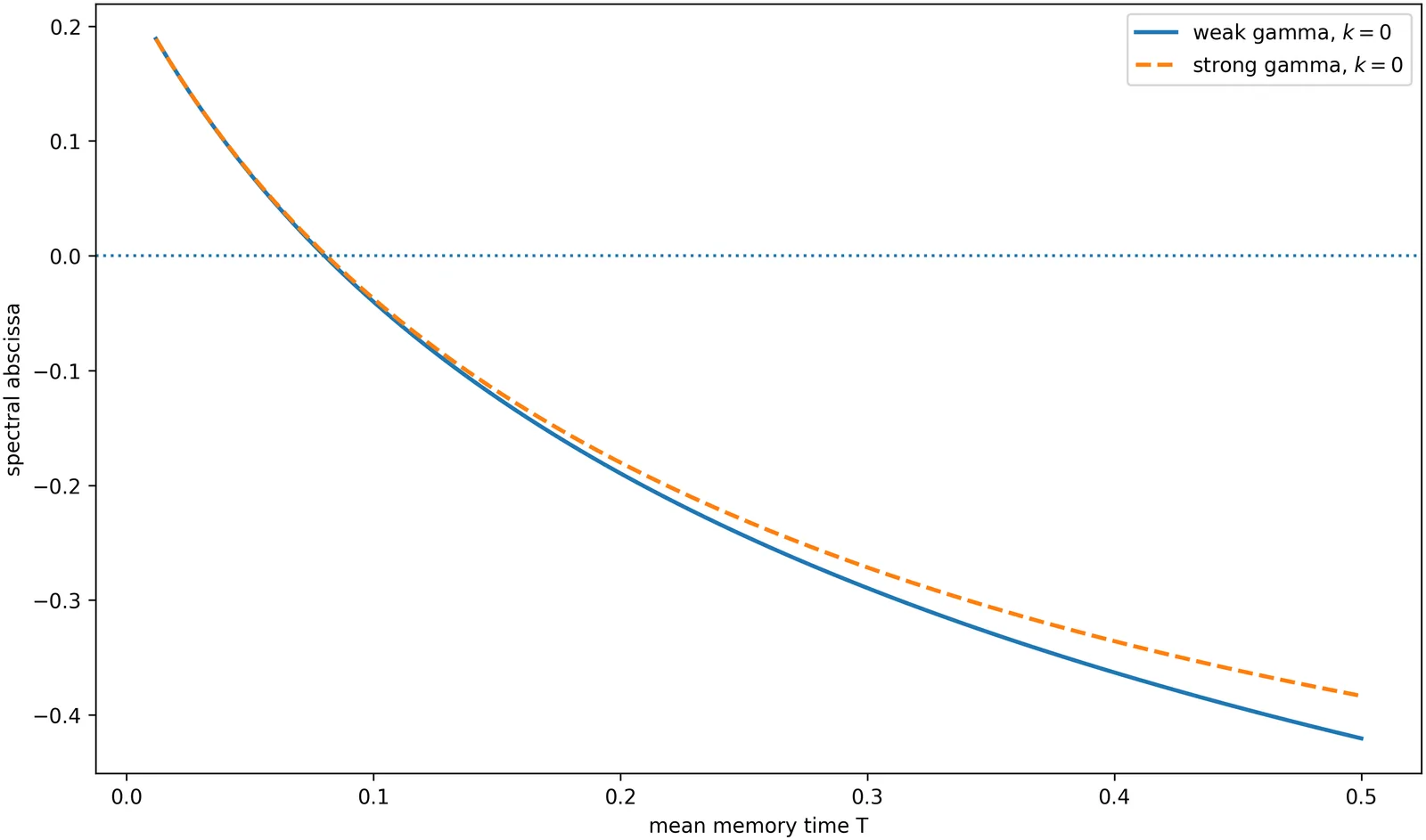
Spectral abscissa for the homogeneous mode (*k* = 0). Largest real part of the characteristic roots versus mean memory time *T* for the reference parameter set. Solid: weak gamma. Dashed: strong gamma. Each curve crosses zero transversally near *T* = 0.08 and is negative for larger *T*; the dotted horizontal line marks zero spectral abscissa. These are stabilising spectral Hopf crossings; no claim of a nonlinear Hopf bifurcation is made from this plot alone.

**Fig 8:**
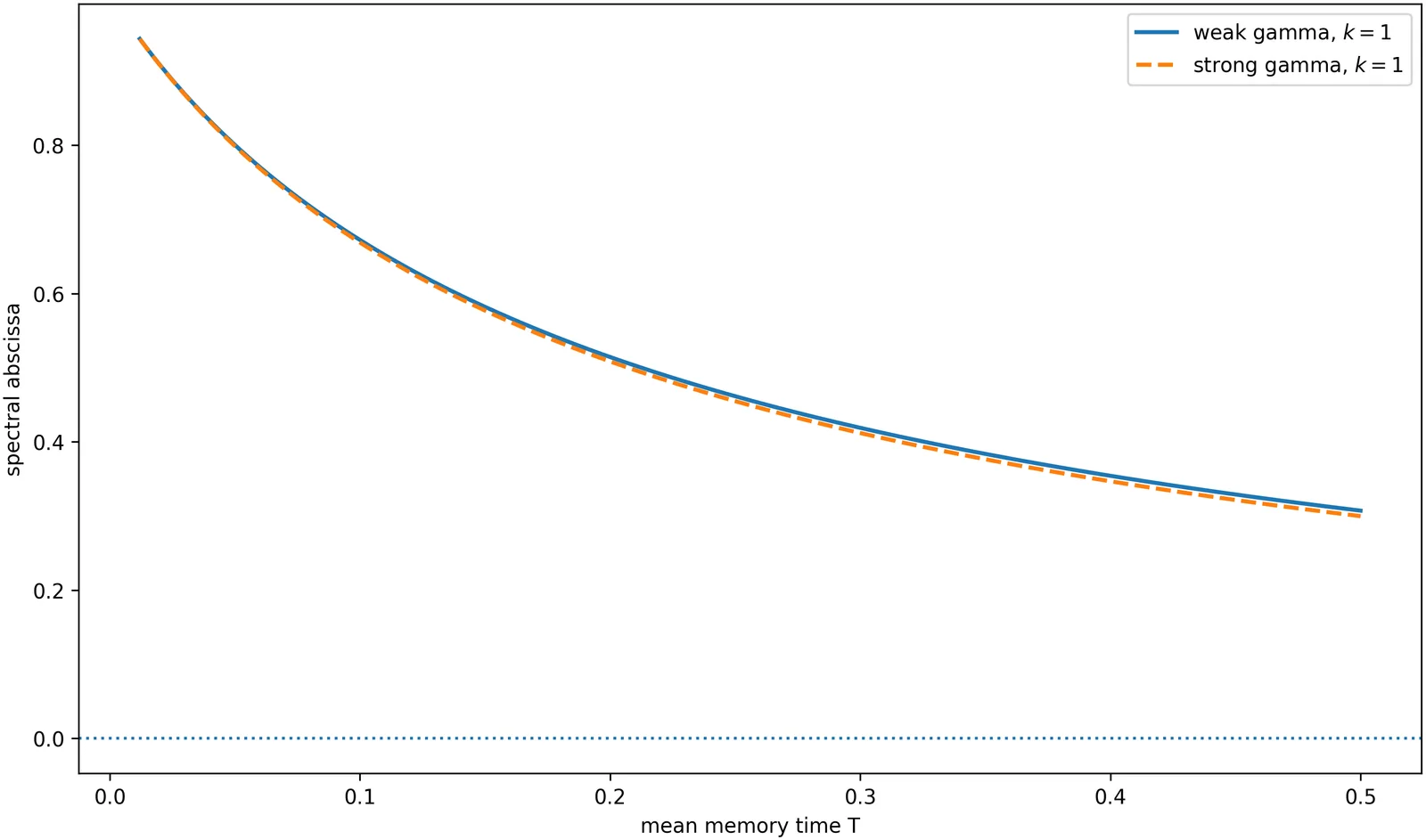
Spectral abscissa for the first spatial mode (*k* = 1). Largest real part of the gamma-model characteristic roots versus mean memory time *T*. Solid: weak gamma. Dashed: strong gamma. Positivity for all *T* > 0 follows from ${𝒞}_{1}<0$ and Proposition 1; hence the instability is memory-independent within both gamma formulations. The dotted line marks zero spectral abscissa.

**Fig 9:**
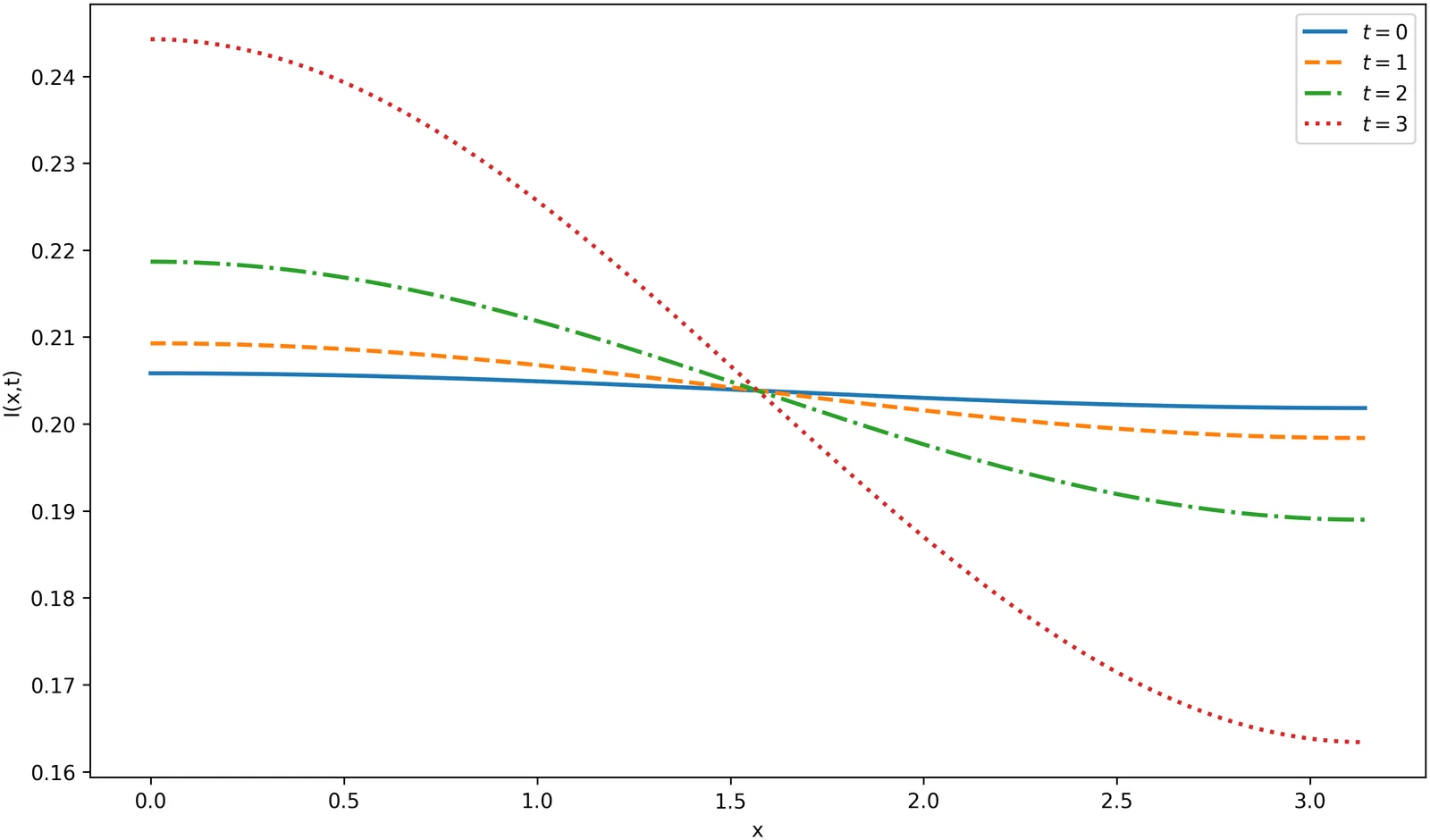
Linear reconstruction of the first Neumann spatial mode. Profiles of $I(x,t)=I^{*}+0.002e^{1.0022t}\cos x$ at *t* = 0,1,2,3 (solid, dashed, dash-dotted and do*tt*ed). This illustrates the positive *k* = 1 zero-delay eigenvalue and is a linear modal visualization, not a nonlinear full reaction–diffusion simulation.

We first recall the benchmark behaviour of the homogeneous reference model. Fig 2 shows the homogeneous discrete-delay dynamics for the two values used to straddle the large reference threshold. When τ=1.2, the delay is below the destabilising value $\tau_{0,d}≃1.335$, and the infected density returns to the endemic level after a damped transient. When τ=1.6, the delay exceeds that threshold and the trajectory develops persistent oscillations. This is the classical interpretation of the reference result: a sufficiently large fixed transmission lag produces a phase lag strong enough to turn the endemic equilibrium into an oscillatory state.

This benchmark is important because it gives the natural expectation one might have before introducing a distributed delay: larger memory times could be expected to favour oscillations. The gamma models show that this expectation is not generally correct. What matters is not only the average length of memory, but also whether the past is concentrated at one point or averaged over a distribution.

We then compare the models at the same nominal memory values. This comparison is useful because it uses the values adopted in the reference simulations, while making clear that the spectral meaning of those values changes across memory laws. To make the comparison fair at the numerical level, we use *T* = 1.2 and *T* = 1.6 as mean memory times in the gamma models, while the reference model uses the corresponding fixed delays τ=1.2 and τ=1.6. These values are far larger than the gamma stabilising threshold $T_{0}≃0.08$. Therefore the gamma systems are expected to be well inside their stable region, whereas the discrete-delay system changes from stable to unstable between the two values.

At the nominal value 1.2, all models converge, but not for the same reason. The discrete-delay model is still below its large destabilising threshold; the gamma models have already crossed their small stabilising threshold and are in the stable regime. Thus the common qualitative outcome, convergence to the endemic level, hides two different spectral mechanisms.

The contrast becomes decisive at the nominal value 1.6. In the reference model, the fixed delay places all feedback at one past time and produces an oscillatory instability. In the gamma models, the same nominal time is only the mean of a distribution; the feedback is spread over a continuum of past states. This averaging attenuates the destabilising phase effect, so the infected density converges instead of oscillating. Figs 3–4 therefore show that the gamma models are not numerical copies of the reference model with a different notation for the delay. They have a different transfer function and a different stability scenario.

After the direct comparison, we focus on the weak gamma threshold itself. This shows the direction of the homogeneous crossing and explains why large mean memories are stable in the weak-kernel model. The previous comparison used large memory values because those are the values used in the reference simulations. Fig 5 instead focuses on the weak gamma threshold itself. The critical value is $T_{0,w}≃0.0802$. For $T_{w}=0.04$, the mean memory time is below this threshold and the homogeneous endemic equilibrium is not stable. For $T_{w}=0.16$, the mean memory time is above the threshold and the solution returns to the endemic level.

The direction of this crossing is the essential point. In the reference discrete-delay model, the large threshold at τ≃1.335 is destabilising: increasing the fixed delay beyond that value creates oscillations. In the weak gamma model, increasing the mean memory beyond *T*_0,*w*_ stabilises the homogeneous mode. Distributed memory therefore acts as temporal averaging rather than as a simple delay that accumulates phase lag.

We then repeat the threshold experiment for the strong gamma kernel. This allows us to see whether changing the shape of the distributed memory changes the first homogeneous transition. Fig. 6 gives the analogous computation for the strong gamma kernel. The critical mean memory time is $T_{0,s}≃0.0810$, very close to the weak-kernel value. The qualitative picture is the same: below the threshold the homogeneous equilibrium is unstable, while above it the solution converges.

The closeness between the weak and strong thresholds suggests that, for the present parameter set, the mean memory time is the dominant quantity controlling the first homogeneous crossing. The shape of the kernel still changes the characteristic polynomial, but it does not substantially shift the first stabilising threshold. This observation is useful in applications: different distributed transmission-memory profiles may have similar local stability effects when their means are comparable.

The time-domain simulations are supported by the spectral abscissa curves, which show directly where the characteristic roots cross the imaginary axis. Fig. 7 plots the largest real part of the homogeneous characteristic roots as a function of the mean memory time. Both gamma kernels cross the imaginary axis near *T* = 0.08 and then remain in the stable half-plane for larger *T*.

This plot explains why the gamma simulations at *T* = 1.2 and *T* = 1.6 are stable: both values lie far to the right of the stabilising crossing. It also explains why the gamma models do not reproduce the large reference threshold. The discrete-delay threshold at τ≃1.335 is generated by a pure phase-shift term, whereas the gamma transfer functions are rational filters and have a different root geometry.

Fig. 8 then separates the spatial mechanism from the homogeneous memory mechanism. For the first spatial mode, the spectral abscissa is positive throughout the memory range considered. This positivity is not created by a Hopf crossing at a positive memory time; it is already present at zero memory because ${𝒞}_{1}<0$.

Thus the first spatial mode should not be interpreted as an instability generated by the gamma memory. The memory may modify its growth rate, but the origin of the instability is the spatial reaction–diffusion balance already visible in the zero-memory characteristic equation.

The last numerical test translates the modal diagnosis into physical space. It visualises how the first Neumann profile grows when the zero-memory spatial eigenvalue is positive. Finally, Fig. 9 visualises the modal statement in physical space. The first Neumann eigenfunction is cosx, and the positive zero-delay growth rate $\lambda_{1,+}(0)\approx 1.0022$ predicts an initial profile growing like $e^{1.0022t}\cos x$. The figure therefore connects the algebraic sign of ${𝒞}_{1}$ with the emergence of a spatially heterogeneous perturbation.

The numerical section therefore leads to a clear interpretation. The homogeneous gamma dynamics are stabilised by distributed temporal averaging, while the first spatial mode is governed by a zero-memory reaction–diffusion instability. These two mechanisms are distinct and should not be conflated when the results are compared with the discrete-delay reference model.

## Comparison with the reference model

The comparison with Song et al. [1] can be summarised at the modelling, analytical and numerical levels.

**Modelling level.** The reference model uses the point value I(t−τ). Its memory operator is therefore concentrated at a single past time and contributes the factor $e^{-\lambda \tau }$ to the characteristic equation. The weak and strong gamma models replace this point evaluation by an average of the past infected density. Their linear transfer factors are α/(λ+α) and $\alpha^{2}/(\lambda +\alpha {)}^{2}$, respectively. This change is not cosmetic: a pure delay mainly introduces phase shift, whereas a gamma memory also filters the feedback by distributing it over past times.

**Analytical level.** In the reference model, the modal characteristic equations are quasipolynomials and Hopf bifurcation is studied through trigonometric equations. In the gamma models, the linear chain trick converts the problem into polynomial characteristic equations. The weak kernel leads to the cubic (7), and the strong kernel leads to the quartic (10). Consequently, stability can be expressed by the Routh–Hurwitz inequalities (11) and (13). The determinants $\Delta_{k}^{\left(w\right)}$ and $\Delta_{k}^{\left(s\right)}$ replace the trigonometric Hopf equations of the discrete-delay model.

**Numerical level.** The numerical comparison is the most important point for interpretation. With the reference parameter set, the homogeneous gamma thresholds are close to *T* = 0.08 and are stabilising. The large discrete-delay threshold used in the reference simulations is instead near τ=1.335 and is destabilising. Therefore using the same nominal values 1.2 and 1.6 does not place the models in analogous spectral regimes: the gamma models are far beyond their stabilising threshold, whereas the discrete-delay model crosses from the stable side to the oscillatory side.

Table 2 also clarifies the role of the first spatial mode. The diagnostic quantity ${𝒞}_{1}$ is negative for the reference parameter set. Hence the first spatial mode is already unstable when memory is absent. The gamma formulation makes this fact visible through the constant term of the modal polynomial. In the discrete-delay formulation the same zero-memory information is less transparent because the characteristic equation is written with the exponential factor $e^{-\lambda \tau }$.

**Table 2:** Conceptual comparison between the reference model and the gamma-distributed-delay extensions.

Model	Memory operator	Modal equation	Main threshold character
Discrete delay	I(t−τ)	quasipolynomial	destabilising at τ=1.335
Weak gamma	$\int_{0}^{\infty }\alpha e^{-\alpha s}I(t-s)\;ds$	cubic	stabilising at *T* = 0.080
Strong gamma	$\int_{0}^{\infty }\alpha^{2}se^{-\alpha s}I(t-s)\;ds$	quartic	stabilising at *T* = 0.081

Overall, the gamma models and the reference model answer different biological questions. The reference model describes a population in which the relevant infectious effect is concentrated after a fixed transmission lag. The gamma models describe a population in which individuals contribute to transmission with heterogeneous transmission-memory histories. The numerical results show that this difference can reverse the stability interpretation: distributed memory smooths and stabilises the homogeneous feedback, while a fixed delay can destabilise it through phase accumulation.

## Discussion

Replacing a fixed transmission lag with a gamma-distributed memory changes the mechanism by which the past infected population affects current transmission. The fixed delay has no averaging effect; it simply sends the feedback through a single time lag. As the lag increases, the feedback can return sufficiently out of phase to generate sustained oscillations. This is the mechanism illustrated by the reference model for τ=1.6.

The gamma kernels behave differently. They distribute the past influence over a continuum of times, and the corresponding transfer functions are rational low-pass filters. This averaging reduces the impact of sharp phase lags and explains why the homogeneous gamma modes become stable once the mean memory time passes the small threshold near 0.08. The simulations at *T* = 1.2 and *T* = 1.6 are therefore not surprising from the spectral point of view: these values are far inside the stable gamma region.

The weak and strong kernels give very close homogeneous thresholds for the chosen parameter set. This suggests that the mean memory time is the leading factor in the first stability transition. Nevertheless, the two kernels are not identical. The weak kernel weights the recent past most strongly, whereas the strong kernel has a two-stage structure and a more concentrated distribution around a positive time. In other parameter regimes, or in nonlinear simulations far from equilibrium, this difference in kernel shape could produce more visible quantitative effects.

The spatial result has a separate interpretation. The sign ${𝒞}_{1}<0$ shows that the first Neumann mode is unstable for every positive α in both gamma models. Thus this mode is better understood as a memory-independent spatial-mode instability rather than as a spectral Hopf crossing generated by the memory law. Because the homogeneous zero-memory mode is itself unstable for the reference parameter set, this is not classified as a classical Turing instability. This distinction is important in applications: temporal recurrence and spatial growth may coexist, but they need not have the same mathematical origin.

From an epidemiological point of view, a distributed timing of transmission-related effects can be more realistic than a fixed lag. Real populations contain variability in exposure, immune response and time to infectiousness [42,43]. The present analysis shows that representing this variability can change not only the numerical value of a threshold, but also the qualitative meaning of the threshold. For the reference parameter set, heterogeneity in transmission-memory timing stabilises the homogeneous dynamics instead of producing the large-delay oscillations associated with a fixed transmission lag.

## Conclusion

This paper introduced weak and strong gamma-distributed transmission-memory kernels into the reaction–diffusion SI model with nonlinear incidence studied by Song et al. [1]. The modification leaves the endemic equilibrium unchanged, because constant histories are averaged into themselves, but it changes the linearised dynamics. The weak kernel gives a cubic characteristic equation for each Neumann mode, while the strong kernel gives a quartic equation. These polynomial forms allow explicit Routh–Hurwitz stability and Hopf conditions. The main conclusion is that the distributed-delay extension has a stability structure different from that of the discrete-delay reference model. For the reference parameter set, both gamma kernels produce a small stabilising homogeneous spectral crossing near *T* = 0.08. By contrast, the reference fixed-delay model has a large destabilising threshold near τ=1.335. This explains why, at nominal memory values 1.2 and 1.6, the gamma models converge whereas the discrete-delay model may oscillate. A second conclusion concerns the first spatial mode. The sign ${𝒞}_{1}<0$ implies instability for every positive α in both gamma models, so its growth is not generated by a positive-memory spectral crossing. Because the homogeneous zero-memory mode is also unstable for the reference parameter set, we do not classify this spatial instability as a classical Turing mechanism. The gamma formulation makes the diagnostic explicit through the constant term of the characteristic polynomial.

Future research may proceed in several directions. A first natural extension is to study more general gamma kernels, with higher shape parameters, in order to understand how the concentration of the transmission-memory distribution affects the stability thresholds. A second direction is to move beyond the local analysis and investigate the nonlinear development of the memory-independent spatial-mode instability, including the possible formation of stable spatial patterns. Finally, it would be useful to test the distributed-delay formulation under different epidemiological parameter regimes, so as to identify when distributed memory stabilises the endemic state and when it may instead amplify oscillatory or spatial effects.

## Supporting information

S1 Data Source data underlying the numerical results reported in the manuscript (CSV format). The file contains the reference parameter set and derived quantities; the homogeneous stability thresholds and critical frequencies reported in Table 1 and Fig 1; the numerical time-series data underlying Figs 2–6; the spectral-abscissa data underlying Figs 7 and 8; and the numerical values used for the spatial-mode reconstruction in Fig 9.
(CSV)
