# Supplementary material for: Gamma–distributed transmission memory in a reaction–diffusion epidemic model with nonlinear incidence
Source: PLoS One. 2026 Oct 14;21(10):e0360508. doi: 10.1371/journal.pone.0360508 (PMC13667834; doi:10.1371/journal.pone.0360508)
Supplement: Supplementary file 4 [file pone.0360508.s005.pdf]

Response to Reviewer #2

We sincerely thank Reviewer #2 for the careful reading of the revised manuscript and for the positive assessment of the changes made in the previous round. We have addressed all the remaining points. The substantive and terminological modifications are highlighted in red in the marked-up revised manuscript, whereas purely typographical corrections to spacing and punctuation have been made without highlighting.

Comment 1.

> *The revised manuscript does a good job of replacing "incubation" with terminology that better describes what the model represents. However, "incubation" still appears in the title and short title, and I recommend changing it there for consistency.*

Response. We thank the Reviewer for pointing out this remaining inconsistency. In direct response to this request, we have changed the title from

> *Gamma–distributed incubation delays in a reaction–diffusion epidemic model with nonlinear incidence*

to

> *Gamma–distributed transmission memory in a reaction–diffusion epidemic model with nonlinear incidence*.

The short title has also been changed to *Gamma-distributed transmission memory* in the submission metadata. In addition, we performed a further consistency check throughout the manuscript and replaced the remaining uses of "incubation" that referred specifically to the mechanism represented by our model. In particular, "discrete incubation delay" has been replaced by "discrete transmission lag", and the description based on "incubation memory/histories" has been replaced by terminology based on "transmission memory" and "transmission-memory histories". The generic reference to incubation delays in the introductory discussion of the broader epidemiological literature has been retained because it is not used there to interpret the memory mechanism of the present model.

Comment 2.

> *The keywords still include "diffusion-driven instability," which it has been agreed it is not the case here.*

Response. We agree and thank the Reviewer for noting this. The keyword *diffusion-driven instability* has been removed from the submission metadata and replaced by *distributed memory*, which is consistent with the terminology and interpretation adopted in the revised manuscript.

Comment 3.

> *The manuscript contains a number of typographical and editorial errors that should be corrected before publication, for example: the duplicated phrase "We then pass to the strong We then pass to the strong kernel" (line 165); "Furthemore" (line 251); the double period at the end of the Abstract; "how the concentration of the transmission memory concentration" (line 421). A careful proofreading pass is recommended.*

Response. We thank the Reviewer for identifying these typographical and editorial errors. All the specific issues mentioned have been corrected:

- the duplicated phrase has been corrected to "We then pass to the strong kernel";
- "Furthemore" has been corrected to "Furthermore";
- the double period at the end of the Abstract has been removed;
- the phrase "how the concentration of the transmission-memory concentration affects the stability thresholds" has been rewritten as "how the concentration of the transmission-memory distribution affects the stability thresholds".

We also carried out a complete proofreading pass to improve typographical, grammatical, and terminological consistency throughout the manuscript. This included correction of residual spacing and punctuation issues, as well as minor editorial refinements that do not affect the mathematical content or the results.

We thank the Reviewer again for these helpful comments, which have improved the consistency and presentation of the manuscript.
