# Supplementary material for: Gamma–distributed transmission memory in a reaction–diffusion epidemic model with nonlinear incidence
Source: PLoS One. 2026 Oct 14;21(10):e0360508. doi: 10.1371/journal.pone.0360508 (PMC13667834; doi:10.1371/journal.pone.0360508)
Supplement: Supplementary file 5 [file pone.0360508.s002.pdf]

The manuscript presents an interesting investigation of reaction–diffusion epidemic models with weak and strong gamma-distributed incubation delays. The analytical framework is generally clear, but several points should be clarified or revised to improve the mathematical consistency, reproducibility, and presentation of the paper. My comments are as follows.

Comment 1: Statement of Proposition 1：

Proposition 1 describes the relevant modal instability as a “zero-memory/zero-delay” phenomenon. However, the proof indicates that when $C_k < 0$, the corresponding mode is unstable for any positive value of the memory parameter, rather than only in the zero-memory case. The authors are therefore advised to revise the statement of Proposition 1 and check whether the related stability statements in the Abstract, numerical analysis, and Conclusions are consistent with this result.

Comment 2: Interpretation of the memory parameters：

The manuscript uses both the memory rate α and the mean memory time T, which vary in opposite directions. The authors should clearly distinguish between increasing α and increasing the mean memory time T. Ambiguous expressions such as “increasing memory” should be avoided or precisely defined.

Comment 3: Transversality condition for Hopf bifurcation：

Theorems 1 and 2 require a transversality condition, but the numerical section only reports the critical parameter values. The authors are encouraged to provide a numerical verification that the relevant derivative is nonzero at each critical point, thereby completing the Hopf bifurcation analysis.

Comment 4: Details of the numerical simulations：

The manuscript should provide additional information about the numerical method, including the time step or error tolerance, initial conditions, initial values of the gamma-memory variables, and the history function used for the discrete-delay model. It should also be clarified whether Figs. 2–6 are obtained from the spatially homogeneous reduced systems or from simulations of the full reaction–diffusion models.

Comment 5: Figure captions and interpretation：

The captions of Figs. 2, 3, 4, and related figures are not sufficiently complete or self-contained. Each caption should clearly identify the model, parameter values, plotted variables, equilibrium level, and the meaning of different curves or line styles. The main text should also explain more clearly how the observed numerical behavior is connected to the stability and Hopf bifurcation results.

Comment 6：The following work should be considered. (Bulletin of Mathematical Biology, 2025, 87:121; Journal of Mathematical Physics. 2025, 66, 042706; Mathematical Biosciences, 2025, 379: 109340; Journal of Mathematical Analysis and Applications, 2026, 557: 130334.)
