# Supplementary material for: Gamma–distributed transmission memory in a reaction–diffusion epidemic model with nonlinear incidence
Source: PLoS One. 2026 Oct 14;21(10):e0360508. doi: 10.1371/journal.pone.0360508 (PMC13667834; doi:10.1371/journal.pone.0360508)
Supplement: Supplementary file 6 [file pone.0360508.s003.pdf]

The manuscript entitled *"Gamma Distributed Incubation Delays in a Reaction-Diffusion Epidemic Model with Nonlinear Incidence"* extends the reaction-diffusion SI epidemic model of Song et al. by replacing the discrete delay with weak and strong gamma-distributed memory kernels and examining the resulting stability and bifurcation structure through a combination of analytical results and numerical simulations. The problem is well motivated, the mathematical framework is clearly presented, and the comparison between discrete and distributed memory formulations provides a useful contribution. However, several aspects of the analysis, interpretation, and presentation would benefit from clarification or revision before the manuscript can be recommended for publication.

1. The Introduction should more fully discuss prior studies that have incorporated gamma- or Erlang-distributed delays in spatial or reaction-diffusion epidemic models. Since the replacement of the discrete delay in the model of Song et al. with weak and strong gamma-distributed memories is presented as a key contribution, it would be helpful to explain more clearly how the present formulation differs from, or extends, existing work. Such context would provide a stronger basis for assessing the novelty and theoretical contribution of the study.

2. The statement in line 70 that the selected kernels are "both biologically interpretable and analytically tractable" would benefit from a more explicit justification of their biological interpretation. The manuscript clearly explains the mathematical structure of the weak and strong gamma kernels and later notes that the weak kernel places the greatest weight on the recent past, whereas the strong kernel represents a two-stage memory with weight concentrated away from zero. However, the biological meaning of these distinctions remains relatively brief. It would strengthen the modelling motivation to clarify how the two kernel shapes correspond to different assumptions about the timing and dispersion of incubation or transmission-related effects, and how the associated mean memory times should be interpreted epidemiologically. This additional discussion would help connect the mathematical formulation more directly to the underlying biological processes.

3. The manuscript should explicitly specify the initial conditions used in the numerical simulations. This is particularly important because the discrete-delay formulation requires an initial history function over the delay interval, whereas the weak and strong gamma formulations require initial conditions for the auxiliary memory variables. Since Figs. 2–6 compare the transient dynamics of these different memory formulations, the manuscript should explain how the history function and auxiliary variables were initialized and clarify whether these choices correspond to equivalent initial memory states across the models. Otherwise, differences observed during the transient phase may partly reflect differences in initialization rather than differences attributable to the memory kernels themselves. In addition, the initial spatial perturbation used in Fig. 9 should be explicitly defined, including

its amplitude and spatial form. Providing these details would improve reproducibility and facilitate a more direct comparison of the model formulations.

4. The manuscript would benefit from greater terminological clarity regarding the interpretation of the memory term. Although the discussion frequently refers to an "incubation" effect, the model is formulated as an SI system and does not include an explicit exposed or latent compartment. Instead, the distributed-memory term modifies transmission through dependence on past infectious states. The manuscript should therefore clarify the biological interpretation of the memory kernel and ensure that terms such as incubation, latency, and infectiousness are used consistently with the mechanism represented by the equations.

5. The manuscript states that, under $A^2\beta > 4d(\mu d)^2$, the model admits a positive endemic equilibrium given by Eq. (5). However, the equilibrium equations appear to yield two positive endemic equilibria under this condition, whereas Eq. (5) reports only one solution branch. The manuscript should clarify whether both endemic equilibria exist and, if so, provide a rationale for focusing on the branch given in Eq. (5). It would also be helpful to specify whether the subsequent stability and Hopf bifurcation analyses apply only to this equilibrium or to both endemic equilibria. If the analysis is intentionally restricted to a single branch, the basis for this restriction should be stated explicitly.

6. The captions of Figs. 3 and 4 state that the gamma models are “stable” or “remain stable.” However, this wording may overstate the result, as the manuscript later shows that stability depends on the spatial mode under consideration. In particular, the subsequent modal stability analysis indicates that, for the reference parameter set, the k=1 spatial mode is unstable for all positive memory values, irrespective of the delay formulation. The threshold T0≈0.08T_0 discussed in connection with these figures refers specifically to the spatially homogeneous mode (k=0). To avoid ambiguity, the captions should clarify that the reported stability refers to the homogeneous mode, or equivalently to stability with respect to spatially homogeneous perturbations, rather than to the full reaction-diffusion system.

7. Following Eq. (17), the manuscript states that “positive roots of (17) that also satisfy (16) are the Hopf values.” This statement appears to be inconsistent with the definitions of the two conditions. Equation (17) is derived from the Hopf condition $\Delta_k^{(w)} = 0$, but condition (16) requires $\Delta_k^{(w)} > 0$ as part of the Routh-Hurwitz stability criteria. It would be helpful to clarify the intended criterion for identifying Hopf bifurcation points.

8. Proposition 1 establishes that when $C_k < 0$, the corresponding mode is already unstable in the zero-memory limit and therefore that the instability is not induced by the memory parameter. However, the interpretation of this instability as "diffusion-driven" would benefit from further clarification. For the reference parameter set, the homogeneous mode (k=0) also

appears to be unstable in the zero-memory case. Consequently, the instability of the k=1 mode alone does not establish a diffusion-driven instability in the classical sense, where diffusion destabilizes an otherwise stable homogeneous equilibrium. The manuscript should therefore clarify the intended meaning of "diffusion-driven" in this context and distinguish it from instability that is merely independent of the memory parameter.

9. The manuscript states that "for $k \geq 2$ the diffusion terms dominate, and all Routh-Hurwitz conditions are satisfied for all positive α, so no Hopf crossing occurs." However, the discussion appears to verify this conclusion only for the case k=2, after which it is extended to all $k \geq 2$ without a corresponding analytical justification. Since the Routh-Hurwitz conditions depend explicitly on k, the manuscript should provide a proof or concise argument showing that these conditions remain satisfied for all $k \geq 2$. Otherwise, the statement should be qualified to reflect the range of modes that has actually been established.

10. The statement that the stabilizing gamma crossings are "the opposite of what happens in the reference discrete-delay model" would benefit from qualification. The manuscript itself notes that the discrete-delay model exhibits a small stabilizing crossing near $\tau \approx 0.08194$, which is comparable to the stabilizing thresholds reported for the weak and strong gamma kernels. Thus, stabilizing crossings are not unique to the gamma-memory formulations. Rather, the key difference appears to be that the discrete-delay model also exhibits a larger crossing near $\tau \approx 1.335$ that is destabilizing, whereas the crossings highlighted for the gamma kernels are stabilizing. Revising the wording to emphasize this distinction would provide a more precise characterization of the results and avoid overstating the contrast between the discrete-delay and gamma-memory models.

11. The manuscript should distinguish more carefully between a Hopf crossing of the linearized characteristic roots and a Hopf bifurcation of the nonlinear system. Theorems 1 and 2 establish the existence of purely imaginary characteristic roots and the associated transversality condition, thereby identifying a spectral crossing. However, the numerical section and several figure captions (e.g., Fig. 7) subsequently refer to these crossings as "Hopf bifurcations." Unless additional results are provided to establish a nonlinear Hopf bifurcation, it would be preferable to distinguish consistently between a spectral Hopf crossing and a demonstrated Hopf bifurcation. This would improve the precision and consistency of the mathematical statements throughout the manuscript.

12. The manuscript is generally understandable, but the numerical section contains several repeated or near-duplicate statements, particularly in the discussion of the benchmark discrete-delay simulations and the subsequent gamma-model comparisons. In addition, some statements describe the models as "stable" even when the analysis or simulations establish stability only for specific spatial modes, usually the homogeneous mode. The manuscript

would benefit from a careful editorial revision to reduce repetition and to ensure consistency in notation, figure captions, and terminology, particularly with respect to terms such as *delay*, *mean memory time*, *stability*, *spectral crossing*, and *Hopf bifurcation*.

13. As a minor presentation point, consider using different line styles (e.g., solid, dashed, and dotted curves) in addition to colors to distinguish the plotted cases in the figures. This would make it easier to identify the individual curves when colors are difficult to differentiate or when the figures are printed in grayscale.
