# Supplementary material for: Gamma–distributed transmission memory in a reaction–diffusion epidemic model with nonlinear incidence
Source: PLoS One. 2026 Oct 14;21(10):e0360508. doi: 10.1371/journal.pone.0360508 (PMC13667834; doi:10.1371/journal.pone.0360508)
Supplement: Supplementary file 9 [file pone.0360508.s004.pdf]

Response to Reviewer 1

We thank Reviewer 1 for the careful reading and constructive suggestions. We have revised the manuscript accordingly.

Comment. Proposition 1 describes the instability as a zero-memory/zero-delay phenomenon, while the proof appears to imply instability for any positive gamma-memory parameter when $\mathcal{C}_k < 0$.

Response. We agree. Proposition 1 has been rewritten as "Memory-independent modal instability." The revised proposition now states that if $\mathcal{C}_k < 0$, then $A_{3,k}^{(w)} = \alpha\mathcal{C}_k < 0$ and $B_{4,k}^{(s)} = \alpha^2\mathcal{C}_k < 0$ for every $\alpha > 0$. Hence the weak and strong gamma models are unstable in that mode for every positive memory rate. For the discrete-delay model we only make the corresponding zero-delay statement, which follows by evaluating the characteristic equation at $\tau = 0$. We also revised the Abstract, numerical discussion, Discussion and Conclusion accordingly.

Comment. The manuscript uses both the memory rate α and the mean memory time T, which vary in opposite directions.

Response. We added an explicit paragraph after the kernel definitions stating

$$T_w = 1/\alpha, \qquad T_s = 2/\alpha,$$

and emphasizing that increasing α shortens the mean memory whereas increasing T lengthens it. Ambiguous expressions such as "increasing memory" were replaced by statements such as "increasing the mean memory time T (equivalently, decreasing α)."

Comment. The transversality condition should be numerically verified at the critical values.

Response. We added explicit numerical checks. For the homogeneous weak-gamma crossing,

$$\left.\frac{d\Delta_0^{(w)}}{d\alpha}\right|_{\alpha_{0,w}} = -8.55138 \neq 0,$$

while for the strong-gamma crossing,

$$\left.\frac{d\Delta_0^{(s)}}{d\alpha}\right|_{\alpha_{0,s}} = -5.63651 \times 10^5 \neq 0.$$

As an additional numerical check, the derivative of the spectral abscissa with respect to mean memory time is approximately -2.14221 for the weak kernel and -2.08672 for the strong kernel. Thus the relevant pair of roots crosses the imaginary axis transversally in both cases.

Comment. More information is needed on the numerical method, time step/tolerance, initial conditions, gamma-memory variables, the history function, and whether the figures come from homogeneous reductions or full reaction–diffusion simulations.

Response. We added a reproducibility paragraph at the beginning of the numerical section. The regenerated Figs. 2–6 use the spatially homogeneous reduced systems and a classical fourth-order

Runge–Kutta scheme with fixed step $\Delta t = 0.005$ on $0 \le t \le 80$. The discrete-delay model is integrated by the method of steps with linear interpolation of the stored delayed state. We use

$$S(0) = S^*, \qquad I(0) = 0.98I^* = 0.1997487373.$$

For the discrete-delay model the history is constant, $I(t) = 0.98I^*$ for $t \le 0$. For equivalent initial memory states we set $V(0) = I(0)$ in the weak model and $V_1(0) = V_2(0) = I(0)$ in the strong model. Figs. 7–8 are spectral calculations, not time integrations. Fig. 9 is now explicitly described as a linear modal reconstruction,

$$I(x,t) = I^* + 0.002e^{1.0022t}\cos x,$$

not as a nonlinear full-PDE simulation.

Comment. Figure captions should be more complete and the link between simulations and stability results should be explained more clearly.

Response. We rewrote the captions of Figs. 2–9. The captions now identify the model, the common parameter set, the plotted variable, the equilibrium level, the meaning of each line style, and the relevant threshold. The surrounding text now distinguishes homogeneous-mode stability from full reaction–diffusion stability and explicitly relates the observed time-domain behavior to the spectral crossings.

Comment. The reviewer suggests considering four recent papers.

Response. We added and discussed all four suggested works in the Introduction as recent context for spatiotemporal, reaction–diffusion and network-based dynamics:

- Zhu and Zheng, *Bulletin of Mathematical Biology* 87 (2025), 121;
- Shi and Zhu, *Journal of Mathematical Physics* 66 (2025), 042706;
- Zhu, Ding and Shen, *Mathematical Biosciences* 379 (2025), 109340;
- Dai, Zhu, He and Dong, *Journal of Mathematical Analysis and Applications* 557 (2026), 130334.

We also clarified that these papers provide broader spatial/network context rather than the same gamma-memory mechanism used here.

We again thank Reviewer 1 for comments that materially improved the mathematical precision, reproducibility and presentation of the manuscript.

Response to Reviewer 2

We thank Reviewer 2 for the careful reading and constructive suggestions. We have revised the manuscript accordingly.

Comment. The Introduction should more fully discuss gamma/Erlang distributed delays in spatial or reaction–diffusion epidemic models and clarify the novelty relative to existing work.

Response. We expanded the Introduction and made the comparison with the closest literature explicit. In particular, we now cite Zhao, Wang and Zhang (2017), who studied a reaction–diffusion epidemic model with a general distributed delay, and Lamrani Alaoui et al. (2022), who analysed a diffusive SEIR model with distributed delay and nonlinear incidence. We also cite Andò, Breda and Gava (2020) for a rigorous epidemiological Erlang-delay/linear-chain treatment. We then state the novelty narrowly: we do not claim to introduce distributed delays or Erlang chains into epidemiology for the first time. Our contribution is the mode-by-mode replacement of the fixed delay in the nonlinear SI reaction–diffusion model of Song et al. by weak and strong gamma transfer operators, followed by a direct comparison of the modal stability structures. The factors $e^{-\lambda\tau}$, $\alpha/(\lambda+\alpha)$ and $\alpha^2/(\lambda+\alpha)^2$ are therefore emphasized as genuinely different operators.

Comment. The biological interpretation of the weak and strong gamma kernels should be made more explicit.

Response. We added a paragraph explaining that the weak kernel puts maximal weight at zero lag and then decays exponentially, whereas the strong kernel has zero weight at the present and concentrates influence around a positive time. We now interpret the kernels as alternative assumptions on the timing and dispersion of transmission-related effects of past infectious states. We also explicitly state that, because the model is SI and has no exposed compartment, the auxiliary variables are memory filters and not latent/exposed compartments.

Comment. The initial conditions and equivalent initial memory states should be specified, including the spatial perturbation in Fig. 9.

Response. This has been addressed in the new numerical-protocol paragraph. The discrete history and the gamma-memory variables are initialized consistently, as described in the response to Reviewer 1. The spatial perturbation in Fig. 9 is now stated explicitly as amplitude $\varepsilon = 0.002$ multiplying the first Neumann mode $\cos x$.

Comment. The terminology "incubation" may be misleading because the model contains no exposed compartment.

Response. We agree. The revised title uses "transmission memory." We also performed a terminology pass through the manuscript: statements that previously described the mathematical memory term itself as an "incubation delay" have been replaced by "transmission lag," "transmission memory," or "transmission-related timing." The model section states explicitly that the SI system has no exposed compartment and that V, V_1 and V_2 are memory filters, not latent/exposed classes. We retain the word "incubation" only when describing biological motivation or terminology in earlier literature, not as a literal compartmental mechanism of the present equations.

Comment. Under $A^2\beta > 4d(\mu+d)^2$, the equilibrium equations appear to have two positive endemic equilibria, while only one branch is reported.

Response. We corrected this. The manuscript now gives both branches:

$$I_\pm = \frac{A\beta \pm \sqrt{A^2\beta^2 - 4\beta d(\mu+d)^2}}{2\beta(\mu+d)}, \qquad S_\pm = \frac{\mu+d}{\beta I_\pm}.$$

All subsequent calculations are explicitly restricted to the upper-infected/lower-susceptible branch used in the comparison with Song et al. For the reference parameters, the second branch is $(S_-, I_-) = (0.5707106781, 0.1533176150)$ and is already a saddle in the homogeneous zero-memory linearization, with eigenvalues approximately -0.4601 and 1.5079.

Comment. Captions describing the gamma models as simply "stable" may overstate the result because stability depends on the spatial mode.

Response. We revised the captions and main text to state "stable with respect to the spatially homogeneous mode" whenever that is what has been established. The regenerated Figs. 2–6 are explicitly identified as simulations of the homogeneous reduced systems. We no longer use these figures to claim full reaction–diffusion stability.

Comment. The sentence after Eq. (17) is inconsistent because Eq. (17) imposes $\Delta_k^{(w)} = 0$, whereas condition (16) requires $\Delta_k^{(w)} > 0$ for stability.

Response. Correct. The sentence has been rewritten. Positive roots of Eq. (17) are now called candidate spectral crossing values. At the crossing we require only $A_{1,k}^{(w)}, A_{2,k}^{(w)}, A_{3,k}^{(w)} > 0$, $\Delta_k^{(w)} = 0$, and $d\Delta_k^{(w)}/d\alpha \neq 0$. The strict stability inequality $\Delta_k^{(w)} > 0$ is explicitly not imposed at the crossing.

Comment. The term "diffusion-driven" should be clarified because the homogeneous mode is also unstable in the zero-memory case, so the situation is not a classical Turing instability.

Response. We agree and changed the terminology. The $k = 1$ behavior is now called a "memory-independent spatial-mode instability." We explicitly state that it is not, by itself, a classical Turing instability because the zero-memory homogeneous mode is not stable. Proposition 1 and the Abstract, numerical interpretation, Discussion and Conclusion were revised consistently.

Comment. The statement for all $k \geq 2$ should be proved rather than inferred from checking $k = 2$.

Response. We agree and have replaced the former numerical inference from $k = 2$ by an analytical proof valid for every $k \geq 2$. Setting $x = k^2 \geq 4$, we obtain

$$p_k = 6x + 2.3294313393, \quad q_k = x + 2.8, \quad \mathcal{C}_k = 6x^2 - 14.4705686607x + 0.9224077500.$$

For $x \geq 4$, $\mathcal{C}_k \geq \mathcal{C}_k|_{x=4} = 39.0401331072 > 0$ and $p_k + q_k - a_{23} = 7x - 0.4705686607 > 0$. Hence all coefficients of the weak cubic are positive. Its Hurwitz determinant is

$$\begin{aligned}\Delta_k^{(w)} = {} & (7x - 0.4705686607)\alpha^2 + (49x^2 + 66.2120387502x + 3.1862503645)\alpha \\ & + (42x^3 + 164.6826074109x^2 + 143.7799588651x + 33.4562427207).\end{aligned}$$

which is strictly positive for every $x \geq 4$ and $\alpha > 0$. For the strong kernel, the quartic coefficients are positive by the same inequalities and

$$\Delta_k^{(s)} = \sum_{m=1}^{5} c_m(x)\alpha^m.$$

The explicit functions $c_m(x)$ are now given in the manuscript. In particular, $c_5(x) = 14x - 0.9411373214 \geq 55.0588626786 > 0$ for $x \geq 4$, whereas $c_1(x), \ldots, c_4(x)$ are polynomials in x with strictly positive coefficients. Thus $c_m(x) > 0$ for all $m = 1, \ldots, 5$, $x \geq 4$, and the strong Hurwitz determinant is also strictly positive for every $\alpha > 0$. Consequently both gamma models satisfy the complete Routh–Hurwitz conditions for every $k \geq 2$ and every $\alpha > 0$, so no spectral Hopf crossing can occur in those higher modes. The value $k = 2$ is retained only as an illustrative numerical example, not as the basis of the proof.

Comment. The contrast with the discrete-delay model should be qualified because that model also has a small stabilising crossing near $\tau \approx 0.08194$.

Response. We revised the Abstract and numerical discussion. The revised text now states that the weak- and strong-gamma thresholds near $T \approx 0.08$ are analogous to the small stabilising discrete-delay crossing near $\tau \approx 0.08194$. The important difference is that the discrete-delay model also possesses a large destabilising crossing near $\tau \approx 1.335$, whereas no corresponding large gamma crossing is found for the reference parameter set.

Comment. The manuscript should distinguish a spectral Hopf crossing from a nonlinear Hopf bifurcation.

Response. We agree. The relevant section is now titled "Stability and spectral Hopf-crossing analysis." Theorems 1–2 are phrased in terms of spectral crossings. We added an explicit caveat that a full nonlinear Hopf bifurcation would require additional nonlinear nondegeneracy calculations, such as a first Lyapunov coefficient on the center manifold. Figure captions and the Conclusion were revised accordingly.

Comment. The numerical section contains repetition and inconsistent terminology.

Response. We edited the section to remove repeated introductory sentences and repeated descriptions of the spectral plots. We also standardized the terminology "discrete delay," "memory rate α," "mean memory time T," "homogeneous-mode stability," and "spectral Hopf crossing."

Comment. Different line styles should be used in addition to colors.

Response. All multi-curve figures have been regenerated using distinct solid, dashed, dash-dotted and dotted line styles. The revised captions identify the corresponding styles so that the figures remain interpretable in grayscale.

We again thank Reviewer 2 for comments that materially improved the mathematical precision, reproducibility and presentation of the manuscript.
